# Infection dynamics, transmission, and evolution after an outbreak of porcine reproductive and respiratory syndrome virus

**DOI:** 10.3389/fmicb.2023.1109881

**Published:** 2023-02-09

**Authors:** Hepzibar Clilverd, Gerard Martín-Valls, Yanli Li, Marga Martín, Martí Cortey, Enric Mateu

**Affiliations:** Department of Animal Health and Anatomy, Universitat Autònoma de Barcelona, Cerdanyola del Vallès, Spain

**Keywords:** porcine reproductive and respiratory syndrome virus, genetic diversity, evolution, founder effect, super-spreader, neutralizing antibodies

## Abstract

The present study was aimed at describing the infection dynamics, transmission, and evolution of porcine reproductive and respiratory syndrome virus (PRRSV) after an outbreak in a 300-sow farrow-to-wean farm that was implementing a vaccination program. Three subsequent batches of piglets (9–11 litters/batch) were followed 1.5 (Batch 1), 8 (Batch 2), and 12 months after (Batch 3) from birth to 9 weeks of age. The RT-qPCR analysis showed that shortly after the outbreak (Batch 1), one third of sows were delivering infected piglets and the cumulative incidence reached 80% by 9 weeks of age. In contrast, in Batch 2, only 10% animals in total got infected in the same period. In Batch 3, 60% litters had born-infected animals and cumulative incidence rose to 78%. Higher viral genetic diversity was observed in Batch 1, with 4 viral clades circulating, of which 3 could be traced to vertical transmission events, suggesting the existence of founder viral variants. In Batch 3 though only one variant was found, distinguishable from those circulating previously, suggesting that a selection process had occurred. ELISA antibodies at 2 weeks of age were significantly higher in Batch 1 and 3 compared to Batch 2, while low levels of neutralizing antibodies were detected in either piglets or sows in all batches. In addition, some sows present in Batch 1 and 3 delivered infected piglets twice, and the offspring were devoid of neutralizing antibodies at 2 weeks of age. These results suggest that a high viral diversity was featured at the initial outbreak followed by a phase of limited circulation, but subsequently an escape variant emerged in the population causing a rebound of vertical transmission. The presence of unresponsive sows that had vertical transmission events could have contributed to the transmission. Moreover, the records of contacts between animals and the phylogenetic analyses allowed to trace back 87 and 47% of the transmission chains in Batch 1 and 3, respectively. Most animals transmitted the infection to 1–3 pen-mates, but super-spreaders were also identified. One animal that was born-viremic and persisted as viremic for the whole study period did not contribute to transmission.

## 1. Introduction

Porcine reproductive and respiratory syndrome virus (PRRSV) is one of the major pathogens of swine. After its emergence in the 1980 decade, the virus disseminated worldwide and, nowadays, this infection has become endemic in most pig-producing countries. The impact of the infection is variable depending on the virulence of the strain and on the presence of other concomitant agents [reviewed in Saade et al. (2020) and Zhao et al. (2021)] but, in general, the costs associated with the disease are significant for the affected farms (Nieuwenhuis et al., 2012; Holtkamp et al., 2013; Renken et al., 2021).

PRRSV is an enveloped, positive single-stranded RNA virus that belongs to the genus *Betaarterivirus,* family *Arteriviridae*, order *Nidovirales* (Brinton et al., 2021). The 15 kb genome encodes for at least 10 open reading frames (ORFs). ORF1a and ORF1b encode for the non-structural proteins (nsp), including the RNA-dependent RNA polymerase (nsp9). ORF2-4 encode for the minor envelope proteins (E, GP2, GP3, and GP4), ORF5 for the major envelope glycoprotein (GP5) and ORF5a protein, ORF6 for the membrane protein (M), and ORF7 for the viral nucleocapsid protein (N) (Snijder and Meulenberg, 1998).

At present, two species of PRRSV are recognized, that had been classified as genotypes until the last taxonomy modification in 2021. *Betaarterivirus suid 1* or PRRSV-1 (formerly called the European genotype) was first identified in the Netherlands in early 1990s (Wensvoort et al., 1991), and *Betaarterivirus suid 2* or PRRSV-2 (formerly designated as the North American genotype) was identified in the United States in 1992 (Benfield et al., 1992).

PRRSV infection causes reproductive failure in pregnant sows and respiratory problems, poor growth rate, and mortality in piglets. After the contact with the virus, susceptible animals develop viremia, that can last from days to months depending on the age; the younger the longer. After the cessation of the viremia, infection persists in lymph nodes for several weeks but finally the infection is cleared. Recovered animals develop strong immunity against the homologous challenge for some months. In contrast, immunity against the heterologous challenge is only partial and infection can take place, although the course is often less severe [revised by Mateu and Diaz (2008)].

The control of PRRSV infection can be achieved by a combination of monitoring, herd management measures, biosecurity programs, and vaccination. Commercial vaccines against PRRSV are made either of attenuated live virus, with or without an adjuvant, or inactivated virus (always with adjuvant). Attenuated vaccines are preferred for the initial immunization of sows while inactivated vaccines are mostly used as recall antigens. In any case, vaccinated animals can be infected since protection is only partial. In sows, the most common vaccination programs start before the first insemination with the aim of protecting them before the first gestation. Afterwards, sows need to be revaccinated every 3–4 months.

Very soon after the discovery of the virus it was evident that PRRSV diversified with a high evolutionary rate. Thus, Forsberg et al. (2001) proposed that, using the ORF3 to set up a molecular clock, the substitution rate was in the range of 4.8 ± 6.9 × 10^−3^ per site per year, a value higher than the estimated one for other nidoviruses, for example coronaviruses (Duchene et al., 2020; Worobey et al., 2020; Ghafari et al., 2022; Tay et al., 2022). This, together with the increasing evidence obtained by sequencing, led to the idea of the ever-expanding diversity of PRRSV (Murtaugh et al., 2010; Shi et al., 2010). In addition, the existence of recombination events further boosts the diversification of the virus (Brar et al., 2014; Martín-Valls et al., 2014).

There are multiple consequences of this genetic diversity. On the one hand, it is known that genetic diversity has an impact on protection. From early studies it was evident that heterologous protection was only partial (Mengeling et al., 2003; Labarque et al., 2004). For example, most strains, induce neutralizing antibodies (Nab) that only significantly neutralize the strain that induced them (Martínez-Lobo et al., 2011). On the other hand, genetic diversity also affects the recognition of T-cell epitopes (Vashisht et al., 2008; Díaz et al., 2009; Parida et al., 2012), and has been related to the immunobiological properties of the strains such as the induced pattern of cytokines (Darwich et al., 2011). Besides this, recombination events have been linked to the emergence of more virulent strains (Shi et al., 2013; Chen et al., 2018; Cui et al., 2022). Moreover, increased genetic diversity and novel glycosylation sites within neutralizing epitopes was observed when vaccination was introduced in a herd (Kwon et al., 2019).

Although the high number of papers dealing with the genetic diversity of the virus, very few have examined how this diversity is generated in the affected farms. It is well known that once the farm is infected, PRRSV most likely will become endemic [reviewed by Pileri and Mateu (2016)]. However, in areas of high density of pig farms, lateral introductions of new strains may happen every few months because of the widespread presence of the virus and the multiple sources of infection. Tousignant et al. (2015) showed that in the United States about one third of the farms suffered a new infection every year. Thus, one of the most common situations is repeated introductions of different PRRSV strains in farms even though sows are vaccinated. This scenario is ideal for the selection of new variants or for the occurrence of recombination events. Within the farm, viral selection and diversification could happen mainly: (1) in the sows, the ones which maintain the infection cycle in the farm through vertical transmission to the offspring, (2) in the piglets, which are infected as soon as they lose the maternally derived antibodies, or (3) in both age groups. This is an almost unexplored area.

The present study explores the genetic diversification of PRRSV over time from the ending of a reproductive disease outbreak until 11 months later. We examined the generation of founder viral variants, tracked transmission chains, assessed the persistence of the variants in the farm, and evaluated the role of the animals born viremic in the evolution of the virus. The characteristics of different viral variants with regards to replication kinetics and the susceptibility to neutralization were also determined.

## 2. Materials and methods

### 2.1. Case farm and timeline

**Figure 1:**
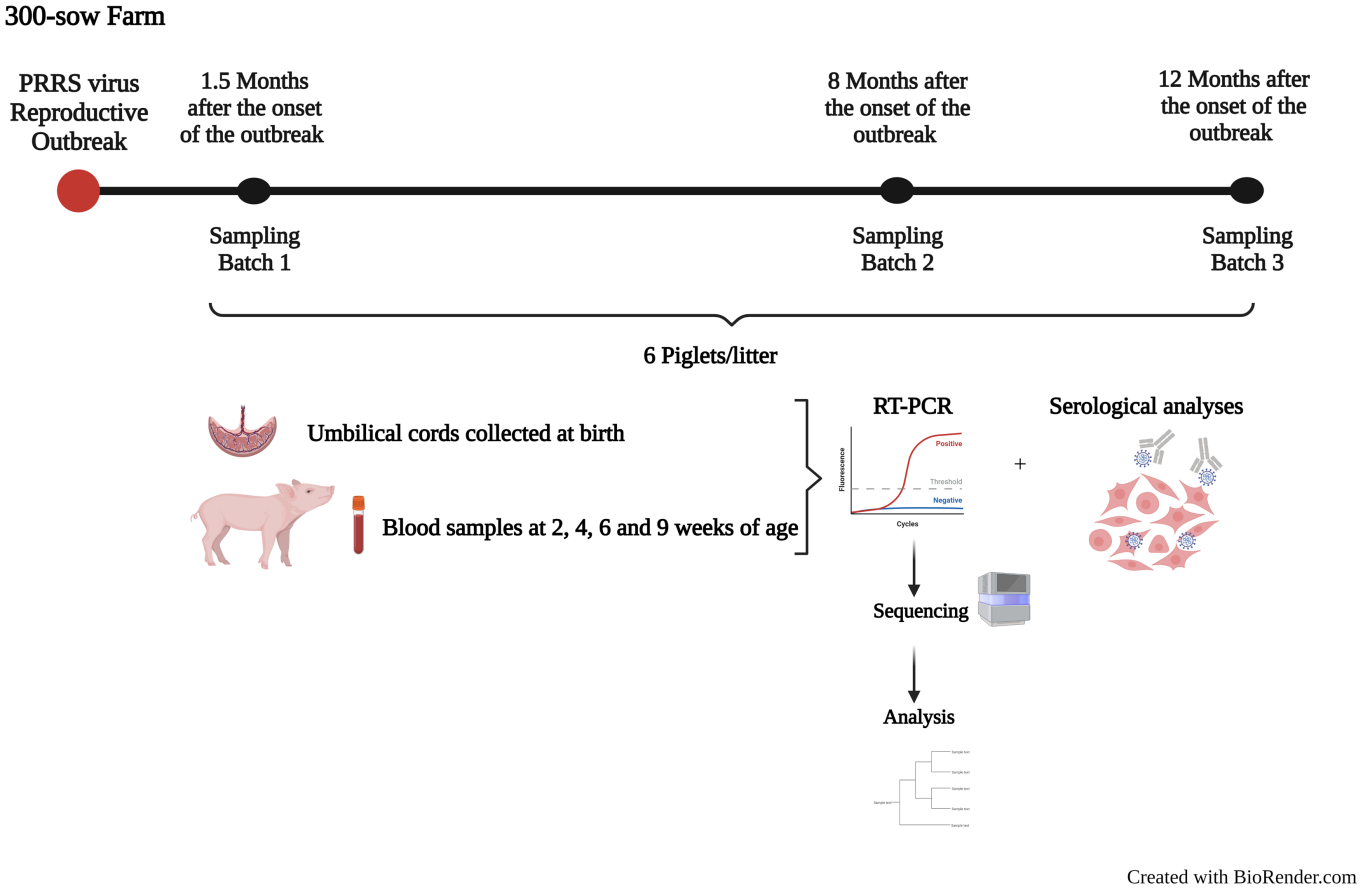
Timeline and design of the present study. The figure shows a summary of the follow-up performed in the farm after the onset of a PRRSV-1 reproductive outbreak. Three batches of piglets were followed 1.5 (Batch 1), 8 (Batch 2), and 12 months after the outbreak (Batch 3) from birth to 9 weeks of age. Samples were used to detect PRRSV by RT-qPCR, sequence, and assess the animals’ serological status against PRRSV.

The examined farm housed 300 breeding sows and raised their offspring until 9 weeks of age, when they were sent to a fattening unit until market-weight age. The farm had been positive to PRRSV for at least 4 years prior to the start of the study. The farm purchased replacement gilts (young sows that have not farrowed or given birth to a litter yet) from a PRRSV-negative herd. Gilts were vaccinated with a modified live PRRSV vaccine (Porcilis^®^ PRRS, MSD, Spain) at least twice before the first gestation and then, together with all sows, received recall doses at least three times per year (blanket vaccination). With this vaccination program the farm had been stabilized and no clinical signs of PRRS were observed for 2 years until a reproductive outbreak occurred. The disease was characterized by abortions, stillbirths, and weak-born animals. PRRS diagnosis was performed by RT-qPCR, but the viral isolate was different from the one circulating before, indicating a lateral introduction of the virus. The timeline and design of the study is shown in Figure 1.

A follow-up of the disease and the viral circulation started 6 weeks after the onset of the outbreak. The first sampling was conducted one and a half months after the onset of the outbreak (Batch 1, 72 piglets from 11 litters), the second one 8 months after the onset of the outbreak (Batch 2, 74 piglets from 9 litters), and the third one 1 year after the onset of the outbreak (Batch 3, 70 animals from 10 litters). At least 6 piglets were selected based on the probability sampling principle within each litter. In each batch, sows of different parity were included (from parity one to eleven overall). The sampling consisted of the collection of umbilical cords (UC) at birth and blood samples at 2, 4, 6, and 9 weeks of age. All animals included in the follow-up were ear tagged to identify them individually.

### 2.2. Detection of porcine reproductive and respiratory syndrome virus by RT-qPCR and viral isolation

Umbilical cords (approximately 4 cm) were collected soon after birth and submerged in a tube containing sterile PBS with antibiotics. Scissors and forceps used for sample collection were decontaminated after each use by submerging them in 2% sodium hypochlorite and were then rinsed with distilled water. The collected UC were minced with sterile scissors and blades, and then the suspension was vortexed and centrifuged at 12,000 × g. The supernatants were aliquoted and stored at −80°C until used. Blood was collected by cava or jugular vein puncture depending on the age of the animal. Serum was separated by centrifugation after clotting and aliquoted to store at −80°C.

Viral RNA was extracted using the NucleoSpin^®^ RNA virus kit (Macherey-Nagel, Germany) according to the manufacturer’s directions, with an elution volume of 50 μL. The presence of PRRSV RNA in the sample was assessed by a commercial RT-qPCR kit aimed to detect both PRRSV-1 and PRRSV-2 (LSI VetMAX™ PRRSV EU/NA kit, Thermo Fisher Scientific, United States) according to the instructions provided. An internal positive control (IPC) was included in each reaction, in addition to positive and negative controls for the RNA extraction and the PCR (samples with a known viral amount and DEPC-water). Samples with C_t_ values ≤37 were considered positive, and between 37.1 and 39.9 were considered inconclusive. With this data, the incidence for each observation period was calculated as the proportion of infected animals over the number of susceptible animals in each time period.

Virus was isolated in porcine alveolar macrophages (PAM) for the subsequent whole genome sequencing. PAM were isolated by bronchoalveolar lavage as reported previously (Mayer and Lam, 1984). To avoid biasing the results, only a single passage was carried out. According to the previous report (Cortey et al., 2018), discrepancies between sample-to-sequence and PAM isolate-to-sequence were 1–3 per 10^−4^ nucleotides with this method. For UC, all positive samples with C_t_ values ≤32.0 were selected for further isolation in PAM. For sera, isolation was attempted from all animals infected at the farrowing units and from their pen-mates that were RT-qPCR positive during the study. Cell culture supernatants of the successfully isolated samples were collected, aliquoted, and stored at −80°C.

### 2.3. Sequencing

For Batch 1 and 2, ORF5, that encodes for the major envelope protein of the virus and has been traditionally used for phylogenetic analyses of PRRSV, was amplified and Sanger sequenced for all positive samples with a C_t_ value ≤32.0 using a previously reported protocol (Mateu et al., 2003). For Batch 3, at least 50% of the samples with the required C_t_ were sequenced for ORF5 (randomly selected). For Batch 1, viral non-structural protein 2 (nsp2) and nsp9 segments, a highly variable and a highly conserved segment of the genome, respectively, were also partially sequenced using tailor-made oligonucleotide primers (Supplementary Table S1).

Whole-genome-based analyses may provide a fuller picture of PRRSV genetic diversity and evolution. Therefore, viral RNA was extracted from the isolates using the TRIzol^™^ reagent (Thermo Fisher Scientific, United States) following the manufacturer’s instructions, with an elution volume of 20 μL. The viral RNA was used for next generation sequencing (NGS) using Illumina Miseq without performing any previous amplification. Briefly, the protocol developed in five steps. First, the genomic library was constructed using a commercial protocol and reagents (Protocol for use with Purified mRNA or rRNA Depleted RNA and NEBNext^®^ Ultra^™^ II RNA Library Prep Kit for Illumina^®^, New England Biolabs, United States). After the NGS run, sequences of low quality were trimmed (QC < 20) using Trimmomatic^©^ (Bolger et al., 2014; RRID:SCR_011848). Then, reads were mapped against a reference sequence (Burrows-Wheeler Aligner applying the BWA-MEM algorithm for long reads; Li and Durbin, 2010; RRID:SCR_010910). The reference sequence was produced from the earliest available isolate obtained during the outbreak by *de novo* assembly using SPAdes^©^ (Bankevich et al., 2012; RRID:SCR_000131). In the fourth step, variant calling was performed with SnpSift^©^ (Cingolani et al., 2012; RRID:SCR_015624) to determine the frequency of each nucleotide at each position of the reference genome. Finally, the viral quasi-species was constructed in fasta format, and the consensus sequence was obtained using Consensus^©^ software, available at www.hiv.lanl.gov/content/sequence/CONSENSUS/consensus.html. The consensus sequences for the whole genomes and the ORF5, the nsp2, and the nsp9 sequences were submitted to GenBank with the Accession Numbers OP688189 - OP688223, OP688224 - OP688357, OP822784 - OP822837, and OP822838 - OP822859, respectively. The NGS raw sequence reads were also submitted to GenBank with the BioProject ID PRJNA915491.

### 2.4. Sequence analyses

For comparative purposes, a selection of available complete genome and ORF5 sequences of PRRSV-1 strains including the five PRRSV vaccines commercially licensed in Spain were downloaded from Genbank (Supplementary Table S2). Phylogenetic trees were constructed using MrBayes (Bayesian inference with 1,000,000 iterations; Ronquist et al., 2012; RRID:SCR_012067) and *p*-distances were calculated using MEGA X (Tamura et al., 2021; RRID:SCR_000667).

For a detailed phylogenetic analysis of the sequences obtained in this study, phylogenetic trees were built up for the whole genome sequence and each viral nsp and ORF using MEGA X (maximum likelihood method, general time reversible model with 1,000 iterations and pairwise deletion) and MrBayes (Bayesian inference with 1,000,000 iterations). P-distances were calculated with MEGA X to determine the range of inter-and intra-batch and clade diversity for the whole genome sequences and the viral segments. The substitution rate was estimated for the whole genome and each segment using BEAST (three independent Markov chain Monte Carlo Bayesian simulations per segment, 10^8^ steps each; Suchard et al., 2018; RRID:SCR_010228). The existence of potential recombination in the whole genome sequences was analyzed using GARD (Pond et al., 2006) and RDP5.0 (Martin et al., 2020). In addition, a comparison of the amino acid composition of the predicted protein sequences was performed between batches, clades, and the vaccine used in the farm.

### 2.5. Transmission chains

Since animals were ear-tagged and all movements of animals between pens were recorded, the chain of infection was tracked through the phylogenetic sequences, pens where animals were located, and the possible direct contacts animals had before the infection. Traceable horizontal transmission was considered to occur when the variant in a newly emerged case was the same as the one presented in a direct-contact pig at the previous observation timepoint. When more than one animal could be considered the source of infection (for example two pigs with the same viral variant had direct contacts), the new case was considered traceable, but the source animal was non-identifiable. With this information, maps of transmission were built, and the incidence of each variant could be estimated approximately.

### 2.6. Intrahost diversity

The viral quasi-species inferred from the NGS runs were used to analyze the distribution and evolution of the intrahost diversity. Among the individuals sampled, two consecutive quasi-species were available for five of them. For every viral quasi-species, the nucleotide frequencies per position obtained in the fourth step of the NGS procedure described in section 2.3 were summarized. The analyses focused on positions with high diversity, arbitrarily defined as those where the frequencies of the mutations present were larger than 10%. Finally, the positions identified were located within the genome.

### 2.7. Viral replication kinetics

To assess the replication kinetics of the different viral variants found in the farm, one isolate was used of each clade. PAM were inoculated at multiplicity of infection (MOI) of 0.01 or medium for negative controls. Three replicates per clade and negative controls were included for each harvest time-point. Supernatants were harvested at 24, 48, 72, and 96 h post-infection. Viral RNA was extracted from the supernatants, and then quantified by RT-qPCR as described in 2.2. An internal positive control was included in all extractions. C_t_ values were plotted against the results of a series of dilutions of the same virus.

### 2.8. Serological analyses

All serum samples in the study were analyzed by ELISA (IDEXX PRRS X3 Ab Test, IDEXX, United States) to determine the animals’ serological status against PRRSV at the moment of infection and the seroconversion if infected. The ELISA results were expressed as S/P ratio values, namely, the ratio between the corrected optical density (OD) of a given sample (OD sample-OD negative control) over the corrected OD of the positive control included in the kit (OD positive control-OD negative control). This S/P ratio allowed semi-quantification and comparison of the results obtained with the different sera. An S/P ratio ≥ 0.40 was considered positive for PRRSV antibody. Additionally, a selection of sera of animals of Batch 1 and 3 was used to assess the capability for neutralizing the predominant viral variant at the end of the study. This selection considered: (i) samples from born-infected animals having viremias longer than 5 weeks, (ii) samples from infected animals at the farrowing units (before weaning) and their PRRSV-negative siblings, (iii) samples from PRRSV-negative animals at 2 weeks of age, (iv) samples taken at 4 and 9 weeks of age of animals infected at 4 weeks of age, or (v) samples from the offspring of sows that were present in the sampling of both Batch 1 and 3 as representative of animals that potentially could have been in contact with the studied viral variants.

Viral neutralization test (VNT) was performed using the protocol described by Yoon et al. (1994) with minor modifications. The virus used for this test was the predominant variant in Batch 3 that had been previously adapted to replication in MARC-145 cells and was fully sequenced following the protocol abovementioned. Only NAb titers ≥2 log_2_ were considered to be relevant. Blood samples of sows (*n* = 96), which were collected after the present study (2–3 months after Batch 3 sampling) for routine health monitoring purposes, were also examined in the VNT.

### 2.9. Statistical analyses

The number of PRRSV vertical transmissions and mortality was compared between batches using a Chi-square test with Yates correction. S/P ratio values at 2 and 9 weeks of age and at 2 weeks of age based on the parity of the sow were compared between batches. Neutralization titers were log_2_ normalized and were compared. All statistical analyses were carried out using GraphPad Prism v9 (RRID:SCR_002798).

## 3. Results

### 3.1. Viral circulation in the farm

**Figure 2:**
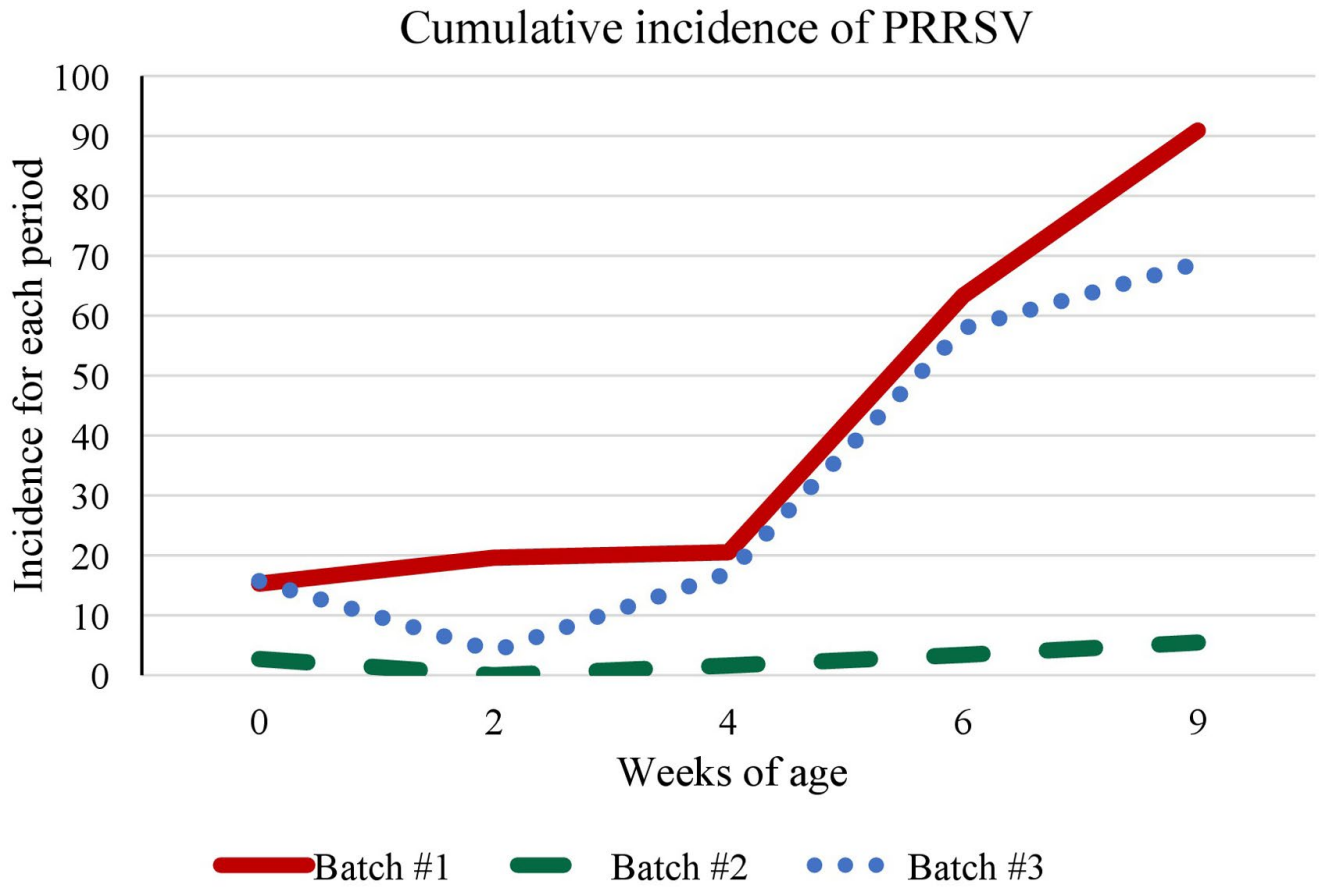
Evolution of the cumulative incidence of PRRSV-1. The graph shows the cumulative incidence of PRRSV-1 for each observation period (2, 4, 6, and 9 weeks of age) per batch. The incidence was calculated as the proportion of infected animals over the number of susceptible animals in a given time period.

Incidence of PRRSV in the different batches is shown in Figure 2. In Batch 1, 11 litters including 72 animals were examined. PRRSV-positive animals were detected in 4/11 litters (36.4%; CI_95%_: 12.4–68.4%) at birth (*n* = 11; between 1 and 5 positive animals per litter; average C_t_ value 30.8 ± 4.0). In 1/11 litter, inconclusive RT-qPCR results (C_t_ > 37.0) were obtained from the UC of the new-born piglets, but infection was confirmed at 2 weeks of age. During the observation period of this batch, 58 animals (80.6%; CI_95%_: 69.2–88.6%) were infected, and 24 (33.3%; CI_95%_: 22.9–45.5%) died in the period between birth and 9 weeks of age, 10 of which in the first 2 weeks of age (41.7% of the total mortality). In Batch 2, viral circulation was very low with only 8/74 animals testing positive overall (10.8%; CI_95%_: 5.1–20.7%, *p* < 0.05) corresponding to two UC and one, two, and three animals at 4, 6, and 9 weeks of age, respectively, with C_t_ values >31.5. Mortality for this batch was 21.6% (CI_95%_: 13.2–33.0%). In Batch 3 (*n* = 70), positive piglets were found in 6 litters at birth (60.0%; CI_95%_: 27.4–86.3%; average C_t_ 35.6 ± 1.0) and results of UC were inconclusive in two other litters. During the observation period, 55 animals (78.6%; CI_95%_: 66.8–87.1%) were infected. Mortality for this batch was 18.6% (*p* < 0.05).

### 3.2. Phylogenetic and evolutionary analyses

The phylogenetic analyses of the sequences obtained in this study together with the PRRSV-1 reference sequences and the five vaccines commercially licensed in Spain indicated that the circulating virus strain in the farm of the present study did not derive from the vaccine used in the farm (Supplementary Figures S1, S2) and had a whole genome similarity of 86.5% to the vaccine strain (Supplementary Table S3). In addition, the analysis showed that variants of the same virus strain were circulating throughout the follow-up and there was no novel introduction of a different virus.

**Figure 3:**
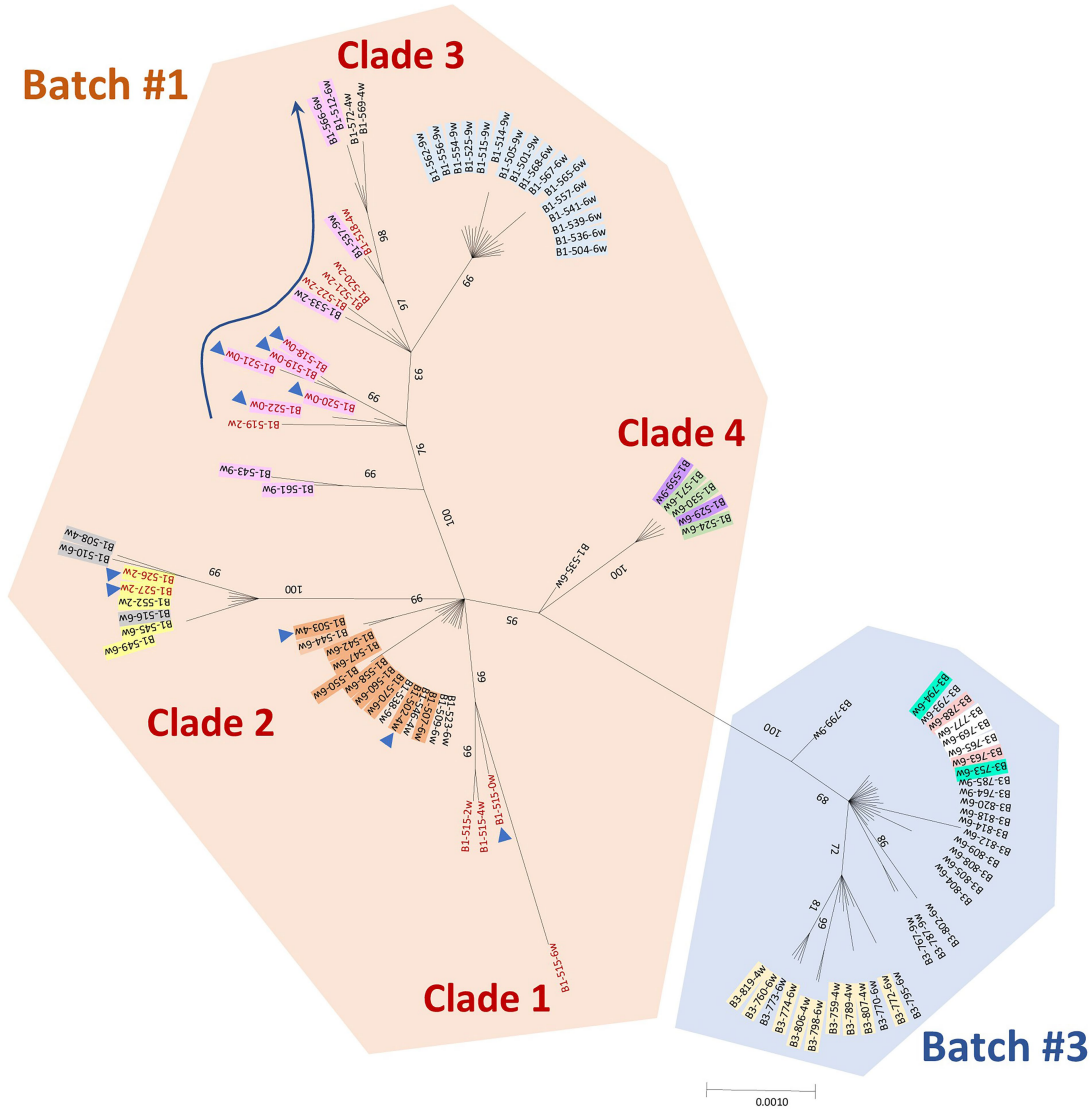
Bayesian phylogenetic tree based on the nucleotide sequences of PRRSV-1 ORF5 (gene size of 606 bp) using MrBayes (1,000,000 iterations). ORF5 sequences obtained from born-infected animals with long viremias (in red) and the new infection cases (in black) are included. The orange and blue shaded areas correspond to sequences retrieved from Batch 1 and 3, respectively. Sequences from vertical transmission events are marked with a blue triangle. The shaded sequences with the same color correspond to animals belonging to a transmission chain. Only posterior probability values >70% are shown.

A Bayesian phylogenetic tree inferred from the ORF5 sequences obtained from born-infected animals and the new infection cases is shown in Figure 3. No quality sequences could be obtained from Batch 2 samples. The phylogenetic analysis of ORF5 showed a clear differentiation of Batch 1 sequences from Batch 3 sequences. In Batch 1, four significantly different clades were distinguished and 3 of them included variants detected in born-infected animals (animal 515 in Clade 1, animals 526 and 527 in Clade 2, and animals 518–522 in Clade 3). Similar results were seen for nsp2 and nsp9 segments (Supplementary Figures S3, S4). The analysis of the whole genome sequences confirmed that Batch 1 and Batch 3 sequences formed two distinct branches (Supplementary Figure S5), and the four abovementioned clades could also be identified in Batch 1. The phylogenetic trees based on maximum likelihood method from ORF5, nsp2, nsp9, and whole genome sequences are shown in Supplementary Figures S6–S9, respectively.

Using ORF5, the net distance between Batch 1 and 3 was 0.0135 ± 0.0041 (Table 1). The estimated annual drift for ORF5 was 1.108% (Table 2). The calculation of the nucleotide mean distance within Batch 1 (*p*-distance 0.0078 ± 0.0020) was 3 times higher than in Batch 3 (*p*-distance 0.0023 ± 0.0009). When the whole genome was used for the calculations of the nucleotide mean *p*-distance within each batch, similar values were obtained (0.0058 ± 0.0004 and 0.0020 ± 0.0002, for Batch 2 and 3 respectively). The estimated annual drift for the whole genome was 0.638%. Furthermore, no recombinant events among the sequences were detected in the analysis.

**Table 1:** Similarities (*p*-distance ± standard error) within and between Batch 1 and 3.

Segment	Batch 1	Batch 3	Batch 1 *vs* 3
**Genome**	0.0058 ± 0.0004	0.002 ± 0.0002	0.0131 ± 0.0011
**ORF1a**			
nsp1a	0.0057 ± 0.002	0.0037 ± 0.0015	0.0155 ± 0.0049
nsp1b	0.0071 ± 0.0018	0.0024 ± 0.0011	0.0127 ± 0.0041
nsp2	0.006 ± 0.0009	0.0025 ± 0.0005	0.0189 ± 0.0025
nsp2TF	0.0068 ± 0.0009	0.003 ± 0.0006	0.0189 ± 0.0022
nsp3	0.0045 ± 0.0015	0.0008 ± 0.0005	0.0104 ± 0.0031
nsp4	0.0088 ± 0.0024	0.0009 ± 0.0005	0.0158 ± 0.0046
nsp5	0.0068 ± 0.0022	0.0021 ± 0.001	0.0141 ± 0.0047
nsp6	0.0102 ± 0.0103	0 ± 0	0.0027 ± 0.0027
nsp7a	0.0092 ± 0.0028	0.0012 ± 0.0007	0.0116 ± 0.0046
nsp7b	0.0033 ± 0.0018	0.001 ± 0.0007	0.0089 ± 0.0046
nsp8	0.0045 ± 0.0024	0.0032 ± 0.0031	0.0039 ± 0.0036
**ORF1b**			
nsp9	0.0042 ± 0.0009	0.0015 ± 0.0005	0.0124 ± 0.0024
nsp10	0.0043 ± 0.0009	0.0015 ± 0.0006	0.0087 ± 0.0023
nsp11	0.0019 ± 0.0008	0.0021 ± 0.0007	0.0118 ± 0.0041
nsp12	0.0077 ± 0.0022	0.003 ± 0.0012	0.0232 ± 0.0064
**ORF2a**	0.0048 ± 0.0014	0.0012 ± 0.0005	0.0053 ± 0.0022
**ORF2b**	0.0033 ± 0.0024	0.0009 ± 0.0008	0.0052 ± 0.0044
**ORF3**	0.0085 ± 0.0021	0.0049 ± 0.0012	0.0076 ± 0.0025
**ORF4**	0.0068 ± 0.0019	0.0017 ± 0.0007	0.0094 ± 0.0037
**ORF5a**	0.0097 ± 0.0056	0.0014 ± 0.0013	0.028 ± 0.0137
**ORF5**	0.0078 ± 0.002	0.0023 ± 0.0009	0.0135 ± 0.0041
**ORF6**	0.0091 ± 0.0025	0.0017 ± 0.0009	0.0174 ± 0.0052
**ORF7**	0.0053 ± 0.0023	0.0009 ± 0.0008	0.0065 ± 0.0036
**3UTR**	0.0283 ± 0.0078	0.0151 ± 0.0067	0.0023 ± 0.0018
**5UTR**	0.0056 ± 0.0025	0 ± 0	0.0185 ± 0.0088

The table shows the nucleotide mean differences within and between the examined batches of PRRSV-1 whole genome consensus sequences and each segment of the viral genome. Nsp, non-structural protein.

**Table 2:** Yearly drift and 95% confidence interval per segment of the viral genome.

Segment	95%-Low	%	95%-High
**Genome**	0.434	0.638	0.867
**ORF1a**			
nsp1a	0.265	0.465	1.029
nsp1b	0.084	0.283	0.627
nsp2	0.229	0.484	0.826
nsp2TF	0.045	0.109	0.197
nsp3	0.311	0.707	1.231
nsp4	0.457	0.966	1.674
nsp5	0.276	1.115	2.352
nsp6	0.000	0.006	1.105
nsp7a	0.122	0.562	1.369
nsp7b	0.183	0.694	1.523
nsp8	0.056	0.501	1.393
**ORF1b**			
nsp9	0.001	0.361	0.863
nsp10	0.351	0.714	1.166
nsp11	0.693	1.391	2.299
nsp12	0.339	1.452	3.091
**ORF2a**	0.272	0.671	1.218
**ORF2b**	0.059	0.475	1.284
**ORF3**	0.265	0.527	0.872
**ORF4**	0.309	0.937	1.748
**ORF5a**	0.983	2.759	5.281
**ORF5**	0.712	1.108	1.568
**ORF6**	0.266	0.887	1.728
**ORF7**	0.164	0.517	1.041

Calculations based on substitution rate estimations obtained by BEAST (Suchard et al., 2018; RRID:SCR_010228), after three independent MCMC Bayesian simulations per segment, 10^8^ steps each, considering the default prior parameters provided by the software.

Amino acid composition differences of the viral proteins between clades of Batch 1 and Batch 3 are shown in Supplementary Table S4. All sequences presented a deletion of 5 amino acids in nsp2 between position 347–351 referred to the prototype PRRSV-1 strain Lelystad (LV; NC043487). There were in total 73 amino acid differences between clades of Batch 1, and 61 differences between Batch 1 and 3. Two amino acid variations in GP4 (amino acid positions 65 and 69) and two in GP5 (amino acid position 41 and 46) affected known neutralizing epitopes (Meulenberg et al., 1997; Plagemann, 2004; Vanhee et al., 2011). Additionally, one of these variations in GP5 (amino acid position 46) was located at a known glycosylation site (Meulenberg et al., 1995; Wissink et al., 2003, 2004). When comparing the predicted amino acid composition of the viral proteins between the virus circulating on the farm and the used vaccine strain (Supplementary Table S5), there were in total 589 amino acid differences. Thirteen amino acid changes in GP4 and 2 in GP5 affected known neutralizing epitopes.

### 3.3. Intrahost diversity

Intrahost diversity was examined at two different timepoints in 5 animals that tested positive for the virus at least twice (2 in Batch 1 and 3 in Batch 3). The results showed that the number of highly variable positions was higher in the quasi-species inferred from the sequences of first batch animals (64) compared to the third batch animals (22.5) (Table 3). Also, the positions were not evenly distributed throughout the genome and the segment corresponding to nsp1a (540 nucleotides) accumulated most of those highly variable positions, 55% for Batch 1 animals and one third for Batch 3 animals. Of note, no significant differences were observed between the first and the second timepoint regarding those highly variable positions in each animal.

**Table 3:** Summary of highly variable positions (positions showing overall mutation frequencies higher than 10%) in the 10 viral quasi-species analyzed, and location of those positions in the PRRSV-1 genome.

Batch	Batch 1				Batch 3					
Animal	518		521		769		770		798	
Age at sampling	4 weeks	6 weeks	2 weeks	4 weeks	6 weeks	9 weeks	6 weeks	9 weeks	6 weeks	9 weeks
**ORF1a**										
nsp1a	29	33	38	27	8	2	7	2	3	3
nsp1b	1	0	0	2	0	0	0	3	1	0
nsp2	17	2	6	9	2	0	1	17	4	7
nsp3	3	0	3	0	0	0	0	3	0	0
nsp4	2	0	1	1	1	0	0	2	0	2
nsp5	4	0	0	2	0	0	0	3	3	0
nsp6	0	0	0	0	0	0	0	0	0	0
nsp7a	6	1	1	0	1	0	0	0	0	1
nsp7b	1	0	0	1	0	0	0	0	1	0
nsp8	1	0	0	0	0	0	0	2	0	1
**ORF1b**										
nsp9	5	1	3	6	0	0	2	4	0	2
nsp10	3	0	1	3	0	0	0	3	0	0
nsp11	0	0	0	0	1	0	1	0	1	0
nsp12	2	0	5	0	0	0	0	0	1	0
**ORF2a**	1	1	1	1	1	1	0	9	8	1
**ORF2b**	0	0	0	0	0	0	0	0	0	0
**ORF3**	1	1	0	1	2	0	1	3	2	1
**ORF4**	0	0	1	0	0	0	0	2	0	1
**ORF5**	5	0	3	4	0	0	0	0	1	2
**ORF5a**	3	0	2	1	0	0	0	0	0	1
**ORF6**	6	0	0	2	1	0	0	0	0	0
**ORF7**	0	0	0	2	0	0	0	0	2	1
Number of highly variable positions	90	39	65	62	17	3	12	53	27	23

The table shows the results for two animals of Batch 1 and three animals of Batch 3 that tested positive in two timepoints.

### 3.4. Transmission chains

The phylogenetic analyses together with the location records of animals at each timepoint led to the identification of possible direct contacts of the infected individuals (shown in Supplementary Material Animation). For Batch 1, the transmission chain was possible to be traced back in 51 out of the 58 infection cases (87.9%), while for Batch 3, 16 out of the 34 (47.1%) sequenced cases. In Batch 1, nine animals were identified as the source of 24 out of 28 horizontal transmission events, and 4/9 of these animals derived from vertical transmission events. In Batch 3, all identified transmissions could be traced to 8 animals. In both batches, most of the identified virus donors transmitted the infection to 1–3 animals; however, in Batch 1, one animal (number 502) was identified as the source of infection for 8 cases, and in Batch 3, one animal (number 777) was identified as the source of infection for 4 cases.

It is worth noting that in Batch 1, 4 animals that were born viremic remained so until the end of the observation period. Of these, three contributed to infect pen-mates but the animal number 515 was not identified as the source of any transmission. Moreover, this animal harbored a viral variant from birth to 6 weeks of age and a different one at 9 weeks of age, suggesting a new infection with a variant horizontally transmitted by another animal.

### 3.5. Viral replication kinetics

**Figure 4:**
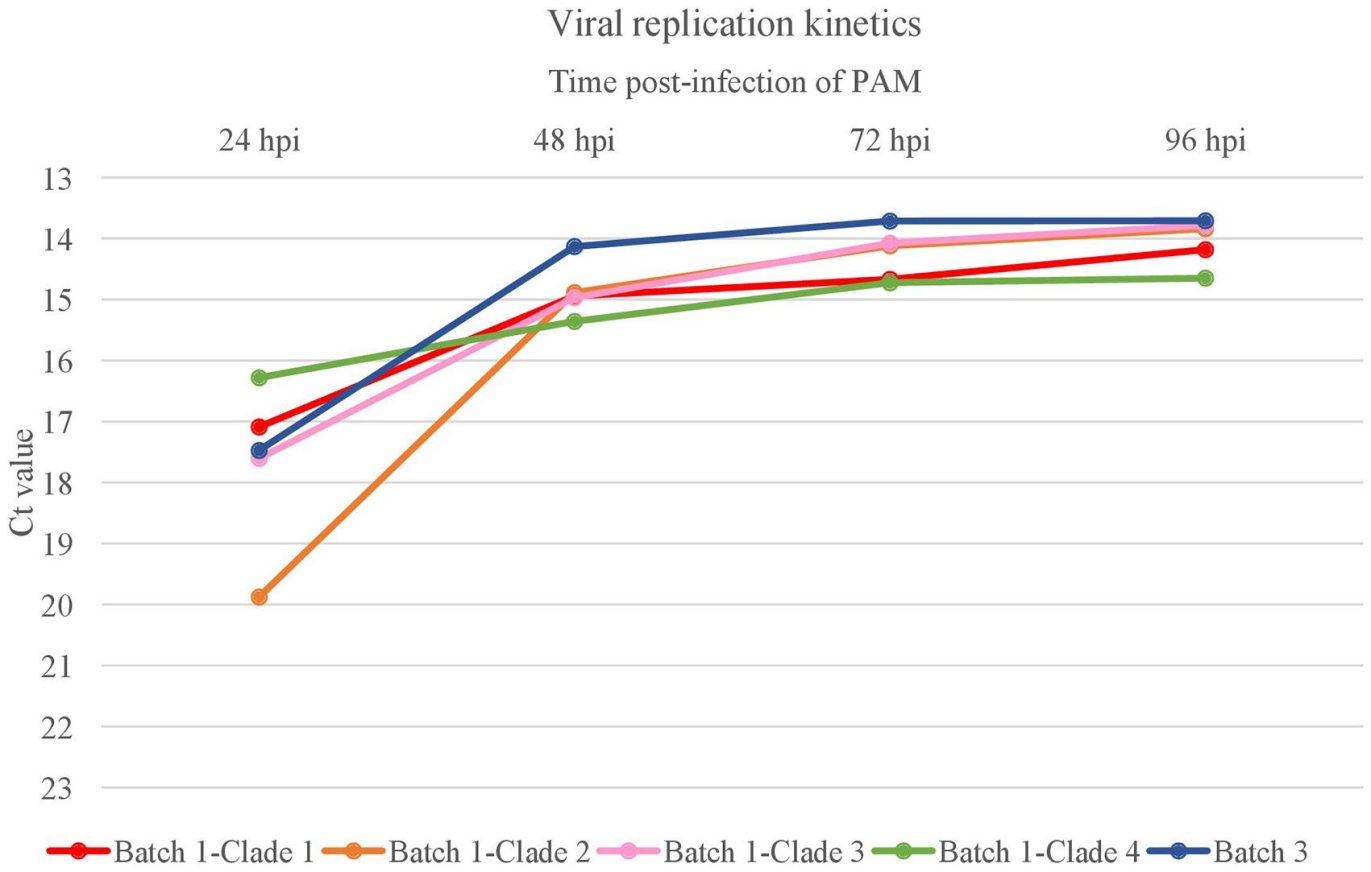
Virus replication kinetics of the different viral clades identified in the farm. The graph shows the kinetic replication curves of variants (*n* = 4) from each clade of Batch 1 and the variant (*n* = 1) in Batch 3 based on C_t_ values of the supernatants harvested at 24-, 48-, 72-, and 96-h post-infection (hpi) of porcine alveolar macrophages (PAM) at multiplicity of infection of 0.01.

To evaluate if differences in transmission were related to the replication capability of the viral variants, replication kinetics were examined for one isolate of each clade (Figure 4). The different clades displayed generally a similar replicative fitness in cell culture. The isolate of Clade 2 might have shown a higher early infectivity, which could suggest a higher affinity to the viral receptors, although it did not result in a higher predominance among the population.

### 3.6. Serological analyses

#### 3.6.1. ELISA antibodies

**Figure 5:**
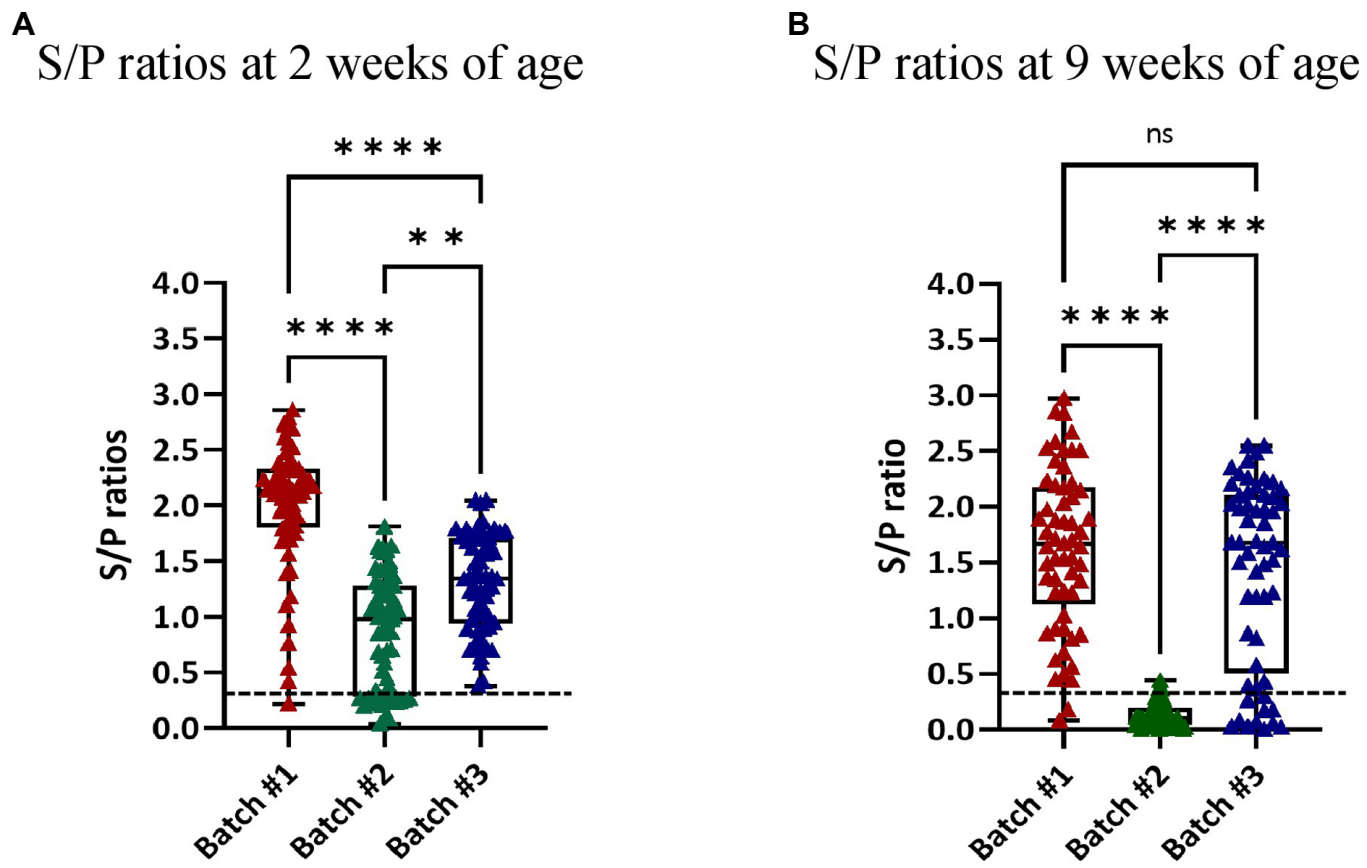
S/P ratios of the piglets as determined by ELISA. Each dot represents an examined individual. S/P ratio values ≥0.4 are considered positive (dotted line). The graphs show the S/P ratio values of the piglets at 2 **(A)** and 9 **(B)** weeks of age in each of the examined batches. ns = not significant.

When the presence of anti-PRRSV antibodies was assessed by ELISA using the sera collected from piglets at 2 and 9 weeks of age, significant differences in the S/P ratio values were noticed. Thus, for 2-week-old piglets, the highest S/P ratios were found in animals from Batch 1, followed by the animals of Batch 3, and the animals of Batch 2 (Figure 5). At 9 weeks of age, S/P ratios were similar between animals of Batch 1 and 3, but almost all pigs in Batch 2 were seronegative, indicating that PRRSV circulation was limited in the nurseries at that time. When the 2-week-old piglets in Batch 2 were examined based on the parity of the sow, the statistical analysis indicated that the offspring of sows ≤6 parity had significantly lower antibody levels (Supplementary Figure S4).

#### 3.6.2. Neutralizing antibodies

The levels of NAb were examined in different groups of animals. Regarding animals that were born-infected (*n* = 7), most of them remained negative all over the observation period and only one was detected as positive at week 6 of age (2 log_2_). The second examined group were those animals that were infected in the farrowing units (*n* = 16) compared to their siblings (*n* = 21). Most of the animals in both groups were negative, indicating NAb were not related to their infection status. Similarly, the examined uninfected animals at 2 weeks of age did not have relevant NAb titers. In addition, average titers at 2 weeks of age were not significantly different when comparing Batch 1 and 3. Next, we examined if animals infected at 4 weeks of age had developed NAb 5 weeks later (*n* = 17). Of the 17 examined animals, only 3 (17.6%) had developed NAb against the circulating strain of PRRSV (one in Batch 1 and two in Batch 3). Figure 5 summarizes the results of the VNT analyses.

In addition, we assessed NAb in the offspring (2 weeks of age) of six sows sampled in both Batch 1 and Batch 3 to ascertain whether maternal-to-fetal transfer of NAb increased with time after potential contact with different viral variants circulating in the farm. Of those six sows, one gave birth to infected piglets in Batch 1, a second gave birth to infected piglets in Batch 3, and a third gave birth to infected piglets in Batch 1 and 3 (Table 4). When the offspring of the sows were examined in Batch 1, positive VNT titers were found in four out of six litters (11/21 animals within the positive litters, 52.4%) with titers ranging from 2 log_2_ to 5 log_2_. When the offspring of the same sows were examined in Batch 3, only 2 litters had positive animals (8/12, 66.7% within the positive litters) with titers ranging from 2 log_2_ to 5 log_2_. It is worth noting that the offspring of 2 of the 3 sows that delivered infected piglets at birth tested negative in the VNT in both Batch 1 and 3. Moreover, the offspring of one sow of parities 9 to 11 (Batch 1 and Batch 3, respectively) always tested negative.

**Table 4:** Parity, PRRSV-1 vertical transmission, and neutralizing antibodies titers of the offspring (2 weeks of age) of six sows that were present in both Batch 1 and Batch 3.

Sow number	Parities	Vertical transmission	N° piglets examined	VNT titers	
	Batch 1 vs. Batch 3	Yes/No, batch	Batch 1 vs. Batch 3	Batch 1	Batch 3
512	9–11	Yes, Batch 3	3–5	Neg.	Neg.
765	4–6	Yes, Batch 1 & 3	4–6	Neg.	Neg.
783	4–6	Yes, Batch 1	4–5	Neg. to 5 log_2_	Neg.
552	9–11	No	4–6	Neg. to 3 log_2_	Neg. to 2 log_2_
635	7–9	No	3–6	2–3 log_2_	Neg. to 5 log_2_
893	1–3	No	5–4	Neg. to 4 log_2_	Neg.

Titers are shown as log_2_ values. Neg., negative.

**Figure 6:**
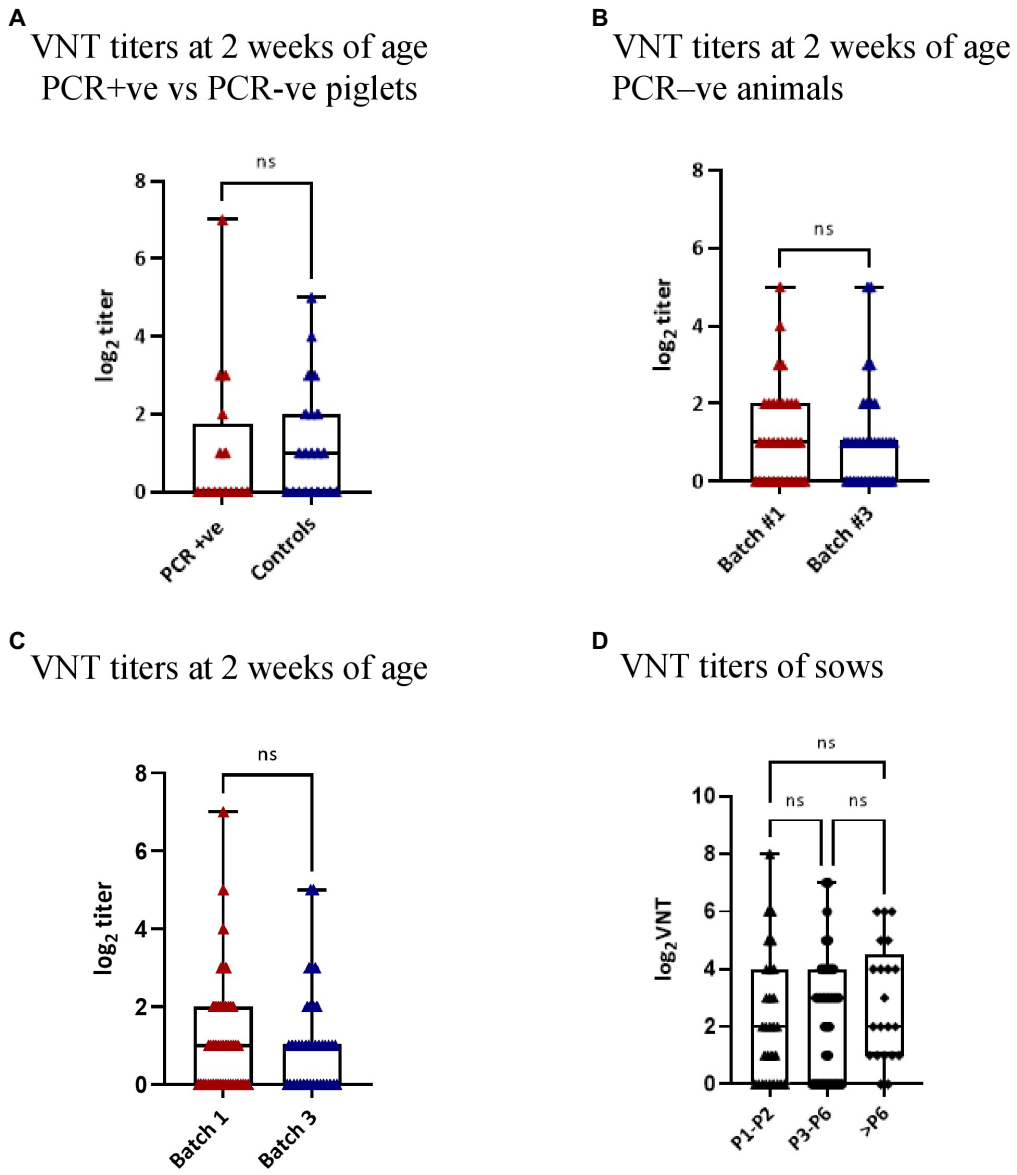
Virus neutralization titers in the different groups. Each dot represents an examined individual. Titers are shown as log_2_ values and only NAb titers ≥2 log_2_ were considered to be relevant. **(A)** The graph compares the VNT titers of the PCR positive (PCR + ve) animals at 2 weeks of age and their PCR negative (PCR-ve) siblings. **(B)** The graph shows the VNT titers of the PCR negative (PCR-ve) animals at 2 weeks of age in Batch 1 and 3. **(C)** The graph shows the VNT titers of 2-week-old piglets of Batch 1 and 3. **(D)** The graph shows the VNT titers according to the parity of the 96 sows sampled one year after the onset of the outbreak. ns = not significant.

Finally, we examined the presence of NAb in sera collected from sows for health monitoring purposes over one year after the original outbreak. At that moment, most of the sows had low VNT titers or were negative (Figure 6) but no differences could be determined regarding parity.

## 4. Discussion

The introduction of PRRSV in a naïve farm usually results in a major reproductive outbreak. After a variable period, the reproductive performance improves, and the farm enters in an endemic state in which the disease is mostly seen in nurseries. It is commonly assumed that the vertical transmission is the key element for the maintenance of the infection in the farm since it results in the birth of infected animals. These piglets can be viremic for a long period and are thought to be efficient transmitters of the disease to their pen-mates. Most of the control strategies for the successful elimination of PRRSV from the farm rely on depopulation or on strategies like the load, immunization, and closure of the farm. In other words, it is assumed that after immunizing all the animals present in the farm and closing the farm to the entrance of new susceptible animals, the circulation of the infection will cease. The duration of the closing period is a key element in those strategies. However, in many farms, depopulation or closure strategies are not economically feasible and other approaches are needed.

In those other cases, monitoring, vaccination, and biosecurity measures are essential for the control. Monitoring usually focuses on the detection of infected animals and the hotspots of viral circulation (replacement gilts, sows, and nurseries). Additionally, it uses sequencing as a tool to determine if the viral circulation in the farm is caused by the previously resident strain or by a newly introduced one. Sometimes the same strain reemerges after a period in which viral circulation seemed to be contained. Our knowledge of the microepidemiology of PRRSV within the farm over time is still limited.

The present study focused on the examination of the diversification of the virus after an outbreak in a vaccinated farm, relating the viral evolution with the variations in the incidence of the infection and the development of an immune response to the virus. For that purpose, we followed a farm for one year after a reproductive outbreak.

We started the study approximately 6 weeks after the initial detection of the outbreak. At that moment about one third of the examined litters had infected animals at birth, indicating a high proportion of susceptible sows. The phylogenetic analysis at that point showed a high viral diversity with 4 recognizable different clades of the virus. Three of these clades were identified in new-borns, suggesting the existence of a founder effect. These founder effects were described initially in HIV and hepatitis C virus and can represent population or selection bottlenecks (Weiner et al., 1993; Gerotto et al., 2006; Russell et al., 2011; Naidoo et al., 2017). In other words, the transmission of the virus from one individual to the other through mucosal surfaces or through the placenta may result in a quantitative selection of viral particles (only few can pass through) or in a qualitative selection (only some viral particles with specific characteristics can be transmitted through the mucosal surfaces). In our case, we cannot know the nature of these bottlenecks but the fact that the sows had been multiple vaccinated and might have been infected previously suggest that both types of bottlenecks could have acted. In a previous work (Cortey et al., 2018) we have shown that selection bottlenecks for PRRSV can exist in vaccinated animals.

After the initial outbreak and the high spread of the infection in the farrowing units and nurseries in the first batch, in Batch 2 (around 6 months later), the viral circulation was limited. The few animals in this batch that tested positive in the RT-qPCR produced very high C_t_ values, indicating low viral loads or even detection of environmental virus for example in the case of UC collection. The key question is why the viral circulation almost stopped. The most intuitive answer would be that the circulation ceased because the population was immune. However, when the animals of that batch were serologically examined, the antibody levels were low in 2-week-old piglets, suggesting that the sows did not have a strong humoral immunity against the virus. Whether this was because of a rapid decline of herd immunity after the outbreak or because of the poor induction of antibodies by that particular virus isolate, or both, is difficult to say. In any case, the absence of antibodies against PRRSV in most of the 9-week-old animals of Batch 2 is a strong indication that the virus was not circulating indeed. Moreover, as we will discuss later, the fact that both piglets and sows had low levels of NAb against the circulating virus suggests that humoral immunity might not be a complete indicator for understanding how immunity affects viral persistence in the herd. Although correlates of protection against PRRSV have not been fully elucidated, Lopez et al. (2007) showed that passive transfer of neutralizing antibodies results in protection in a homologous challenge model when titers of 1:8–1:16 are reached. However, there is evidence that cell mediated immunity also plays a role in protection against PRRSV-1 infection in the homologous and heterologous models (Díaz et al., 2012).

Then, we examined Batch 3 four months after Batch 2. Surprisingly, the infection not only did not disappear but re-emerged, and at the end of the nursery period most animals had been infected. How did this happen? The phylogenetic analysis of Batch 3 isolates clearly showed that a new variant of the resident virus gained predominance. However, the variant found at this moment was much less diverse than any variant found in Batch 1, indicating a strong selection process that happened very close to the timepoint when Batch 3 was examined. This could reflect the selection of a variant that was able to escape the immune response and that was transmitted efficiently in the vaccinated population that had been infected or in contact with the initial one. One evident explanation could have been the selection of a variant with enhanced replication or capacity of transmission. Our replication kinetics experiments however did not support the hypothesis of an enhanced replication capability, although we cannot discard higher potential for transmission, not caused by an increased yield of replication itself, but by a better replication in the nasal mucosa macrophages (Frydas and Nauwynck, 2016).

Interestingly, in Batch 3 the proportion of vertical transmission events was very high suggesting again that either immunity against the new variant was not strong enough in the population or that non-immune sow subpopulations existed. When we examined the NAb against the variant predominating in Batch 3, it was evident that even sows that had been infected (as demonstrated by the delivery of infected piglets) developed very low levels of NAb. This is an indication that the strain present in the farm was probably a poor inducer of NAb. Moreover, the fact that the offspring of old sows that had been vaccinated on multiple occasions and had given birth to infected piglets were negative in the neutralization tests indicates the vaccination did not contribute to enhance the humoral response of the sows against an unrelated strain. Furthermore, it is evident that subpopulations of susceptible sows can be found in multiply vaccinated farms with high circulation of wild type virus (Díaz et al., 2020; Fiers et al., 2022). In a small farm like the one studied here, after an outbreak and with vertical transmission rates of 30%, it seems unlikely that solely by chance so many sows did never encounter the wild-type virus and, in consequence, did not produce antibodies specific for the farm strain. It is tempting to think that they are unresponsive sows. This hypothesis would be supported by the observation that the offspring of sows that transmitted the infection at birth did not have Nabs, not even in the case of an old sow delivering infected piglets twice. These breeders are clear candidates to maintain infection on the farm. Elucidating the role of those poor responder sows and determining if the lack of response occurred from the initial immunization or was the result of receiving multiple vaccine doses would be important to understand how the virus may persist in vaccinated farms. However, the fact that the reproductive disease was under control after the initial outbreak, and no changes in the farm management or the vaccination schedule were performed, suggests that some immunity existed in sows. If NAb specific for the farm strain were low, the most logical explanation would be that cell-mediated immunity had a role in this control. However, the fact that in Batch 3 vertical transmission was high opens the question of the duration of the efficacious immunity after infection with some strains.

Taken together, in our opinion, the most likely scenario was as follows: the introduction of a new strain in the farm found a population that had limited immunity against that strain despite vaccination. At that moment, the infection spread rapidly, affected pregnant sows, and they transmitted the infection vertically to the offspring. This vertical transmission involved several bottlenecks (transmission through placenta, transmission to vaccinated sows) that generated, from the replication of founder variants, a higher diversity circulating in the piglets, a fact that suggested that the bottleneck selection had a component of probability derived from different phenomena (population bottlenecks, genetic bottlenecks, etc.). In a subsequent phase, the population became immune and limited the transmission to a few individuals. However, since the strain infecting the animals in the farm apparently was a poor inducer of immunity (low levels of NAb and rapid decay of these), susceptible subpopulations rapidly appeared in which the virus kept circulating until a fitter variant was selected and spread again in the population. At this point, all previous diversity would disappear and would be replaced by the new variant. An interesting question raises here, is it possible to identify beforehand what animals contribute to the maintenance of the virus in that phase of limited circulation? This is an area that needs to be explored in further studies.

Interestingly, when highly variable positions were examined within the viral quasi-species of animals in Batch 1 and 3, the number of highly variable positions decreased in Batch 3, suggesting that selection reduced the variability in those highly variable positions. Furthermore, in both Batch 1 and 3, a high proportion (30 to 55%) of the highly variable positions within the quasi-species were in nsp1a despite that nsp1a only accounts for roughly 3.5% of the viral genome. Nsp1a is involved in the downregulation of type 1 interferon (Beura et al., 2010; Chen et al., 2010; Beura et al., 2012). This activity has been located in the PCPα domain of the protein. Of note, almost all the highly variable positions in nsp1a were found outside that area, in the zinc-finger motif, suggesting that variability was not related with the inhibition of the interferon response. Besides, nsp1a has been shown to contain at least two epitopes inducing IFN-gamma responses in CD8 T cells (Mötz et al., 2022). One of the most highly variable positions affected one of them but it is difficult to predict the result of this variability on the effective immunity against the virus. Moreover, nsp1a is involved in the induction of apoptosis, pro-inflammatory responses, and the regulation of minus strand templates (Nedialkova et al., 2010; Wang et al., 2016; Park et al., 2021) and thus the variability may respond to other functionalities of the protein.

The present study also provides some relevant information about transmission. The identification of animals, viral variants, and movements between pens allowed to determine, at least partially, the transmission chains. These data showed that while most infected animals contributed to 1–3 transmissions, some animals apparently did not transmit the infection to anyone else, and one (animal 502) was identified as the source of 8 cases out of 28 horizontal transmission events (28.6%) behaving like a super-spreader. Whether this superspreading capability derived from host characteristics, environmental conditions, or both, cannot be determined. It was recently suggested that superspreading events can be an intrinsic characteristic of each viral infection (Chen et al., 2021). It is not known if different viral variants may be more prone to produce superspreading events, but, in our opinion, this would not be the case in our study since other animals infected by the same variant transmitted the infection only to one susceptible recipient.

Another surprising observation was the fact that one animal that was born-viremic and persisted as so for the whole length of the study, apparently did not contribute to the transmission of the virus. In general, it is thought that animals infected with PRRSV *in utero* are important spreaders due to the long viremias they develop (Rowland et al., 1999; Benfield et al., 2000; Rowland et al., 2003). However, the evidence provided by this study indicates that, at least in some cases, these animals may not contribute significantly to the transmission. It would be interesting to examine the shedding of the virus in oral fluids and nasal secretions of these born-viremic animals compared to animals infected after birth to determine differences in transmission capabilities.

From the practical point of view, it is also worth commenting the results of Batch 2. Commonly, PRRSV stability is determined after the examination by RT-qPCR of a number of animals at weaning (usually 30 to 60 animals at wean, in four consecutive batches; Holtkamp, 2011). In our case, only one sample out of 62 (1.6%) was positive at weaning with a C_t_ of 31.9. This suggests that in endemic situations sampling to determine stability of a herd should be of at least 60 animals, and probably more. The low prevalence at weaning in that batch was enough to allow the reemergence of the virus some weeks later.

## 5. Conclusion

The present paper reports an example of how PRRSV may persist in a vaccinated farm. Upon introduction of a new strain, founder effects related to transplacental transmission and high rates of horizontal transmission produce a high diversity of viral variants, but it simultaneously generates an immune or partially immune population, resulting in a low-level circulation of the virus. The combination of subpopulations that just by chance did not have contact with the virus together with a viral strain that was a poor inducer of long-lasting immunity, kept the virus circulating at a low level. Nevertheless, this was enough to allow the selection of a variant that became predominant afterwards. The role of super-spreaders and long-term viremic animals must be re-examined after the results of the present study.

## Data availability statement

The datasets presented in this study can be found in online repositories. The names of the repository/repositories and accession number(s) can be found in the article/Supplementary material.

## Ethics statement

The animal study was reviewed and approved by Ethics Committee in Animal and Human Research of the Universitat Autònoma de Barcelona. Written informed consent was obtained from the owners for the participation of their animals in this study.

## Author contributions

MM, MC, and EM designed the study. Laboratory work was performed by HC, YL, GM-V, MC, and EM. EM, MC, GM-V, YL, and HC analyzed the results. EM, MC, and HC prepared the draft of the manuscript. All authors have read, revised, discussed, and agreed to the published version of the manuscript.

## Funding

This study was funded by the Spanish Ministry of Economy and Competitiveness (project AGL2017-87073-R). HC is supported by a pre-doctoral fellowship *Ayudas para la formación de profesorado universitario* (FPU) of the Spanish Ministry of Universities (grant number FPU18/04259). MC was supported by a *Ramón y Cajal* contract of the Spanish Ministry of Economy and Competitiveness (grant number RyC-2015-17154).

## Conflict of interest

The authors declare that the research was conducted in the absence of any commercial or financial relationships that could be construed as a potential conflict of interest.

## Publisher’s note

All claims expressed in this article are solely those of the authors and do not necessarily represent those of their affiliated organizations, or those of the publisher, the editors and the reviewers. Any product that may be evaluated in this article, or claim that may be made by its manufacturer, is not guaranteed or endorsed by the publisher.
