# Supplementary material for: Infection dynamics, transmission, and evolution after an outbreak of porcine reproductive and respiratory syndrome virus
Source: Front Microbiol. 2023 Feb 9;14:1109881. doi: 10.3389/fmicb.2023.1109881 (PMC9947509; doi:10.3389/fmicb.2023.1109881)

Supplementary Material

Table S1. List of oligonucleotide primers (F: forward primer, R: reverse primer) used for amplifying PRRSV-1 ORF5, nsp2, and nsp9 in this study and the size of the amplicon.

Gene		Sequence	Amplicon (bp)
**ORF5**			
	**L1F (F)**	5′-TGAGGTGGGCTACAACCATT-3′	702
	**L1R (R)**	5′-AGGCTAGCACGAGCTTTTGT-3′	
**nsp2**			
	**nsp2-F1 (F)**	5′-AGGAGTTTTGGACCCTTGACA-3’	617
	**nsp2-R1 (R)**	5′-GGGTCAARTCCAGTGGTTC-3’	
	**nsp2-F2 (F)**	5′-ACAGAGACTTGCCCTCCTCA-3’	643
	**nsp2-R2 (R)**	5′-GCACTGTTCATATACCCGGC-3’	
	**nsp2-F3 (F)**	5′-GCCGGGTATATGAACAGTGC-3’	678
	**nsp2-R3 (F)**	5′-AAAACACCCAGACGAACACG-3’	
**nsp9**			
	**nsp9-F (F)**	5’-TGACCACTGAACAGGCTTTAAACT-3’	684
	**nsp9-R (R)**	5’-CATAAAAGGAGTGTCAGGGCG-3’	

Table S2. List of PRRSV-1 complete genome sequences retrieved from GenBank used for the phylogenetic analysis. Lelystad virus is the prototype for PRRSV-1. Contemporary strains within the same geographical zone of the farm (underlined) and the 5 commercially licensed vaccines in Spain (in red) are included.

Accession Number	Strain ID	Country	Year
NC043487	***Lelystad virus***	Netherlands	1993
KF203132	*Olot/91*	Spain	1991
JF276431	*CReSA3262*	Spain	1992
KX249748	*CReSA3*	Spain	2013
KX249755	*CReSA228*	Spain	2013
KX249756	*CReSA261*	Spain	2013
KX249753	*CReSA100*	Spain	2014
OP822977	*Nu4a*	Spain	2021
OP822964	*M3*	Spain	2021
OP822963	*N5*	Spain	2021
OP822973	*Nu1*	Spain	2021
OM893828	*R1*	Spain	2021
OM893829	*R2*	Spain	2021
JF276435	*CReSA3267*	Portugal	2006
KT326148	*AUT13-883*	Austria	2013
JF802085	*Lena*	Belarus	2007
KP889243	*SU1-Bel*	Belarus	2010
GU737264	*07V063*	Belgium	2007
KT159248	*13V091*	Belgium	2013
KT159249	*13V117*	Belgium	2013
GU047344	*BJEU06-1*	China	2006
MN927227	*HeB3*	China	2018
KC862567	*DK-2011-0511-14*	Denmark	2011
KY767026	*FR-2014-56-11-1*	France	2014
MH018883	*FR-2016-56-11-1*	France	2016
MH463457	*HU19401*	Hungary	2016
MF346695	*PR40-2014*	Italy	2014
KX668221	*WestSib13*	Russia	2013
KY434183	*CBNU0495*	South Korea	2016
GU067771	***Unistrain®PRRS***	-	-
KJ127878	***Porcilis® PRRS***	-	-
LQ787782	***Suvaxyn® PRRS***	-	-
GQ461593	***PYRSVAC-183®***	-	-
KT988004	***PRRSFlex®EU***	-	-

Figure S1. Bayesian phylogenetic tree based on complete genomes of PRRSV-1 using MrBayes (1,000,000 iterations). The orange and blue shaded areas correspond to sequences retrieved from Batch 1 and 3, respectively. Lelystad strain is the prototype for PRRSV-1. The red colored strains are the vaccines commercially licensed in Spain. The underlined strains are contemporary strains within the same geographical zone of the farm. Only posterior probability values >70% are shown.

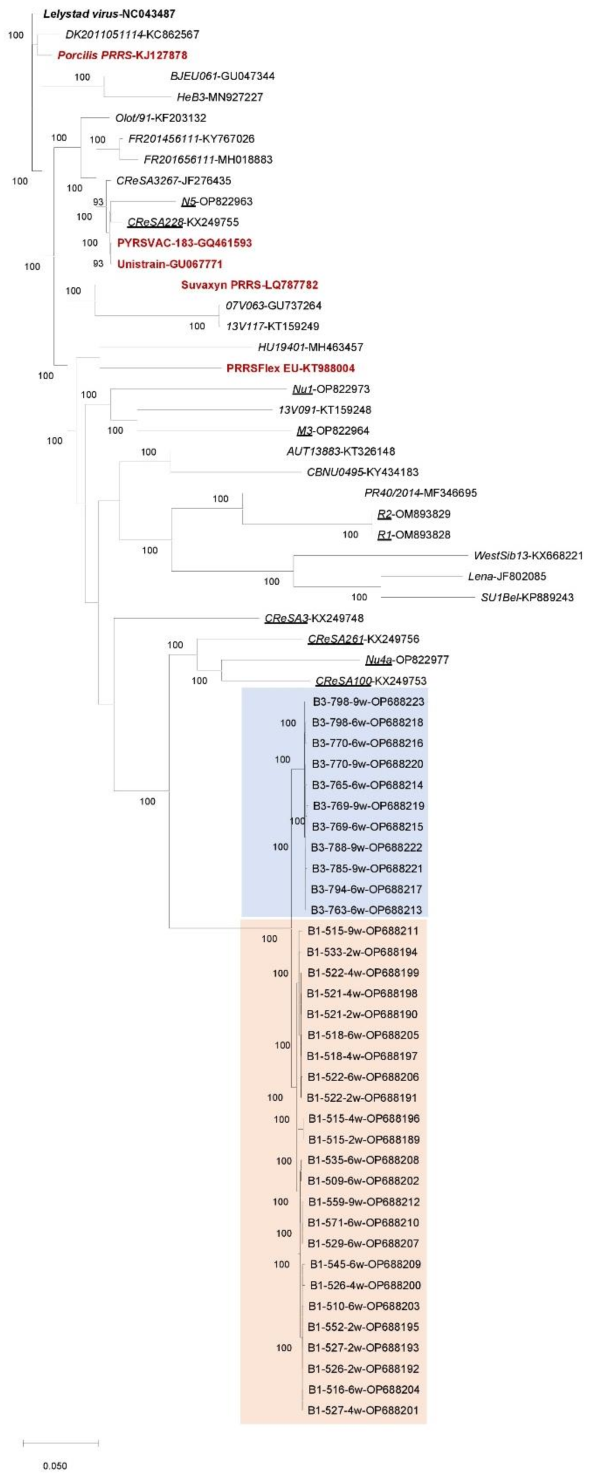

Figure S2. Bayesian phylogenetic tree based on PRRSV-1 ORF5 sequences using MrBayes (1,000,000 iterations). The orange and blue shaded areas correspond to sequences retrieved from Batch 1 and 3, respectively. Lelystad strain is the prototype for PRRSV-1. The red colored strains are the vaccines commercially licensed in Spain. The underlined strains are contemporary strains within the same geographical zone of the farm. Only posterior probability values >70% are shown.

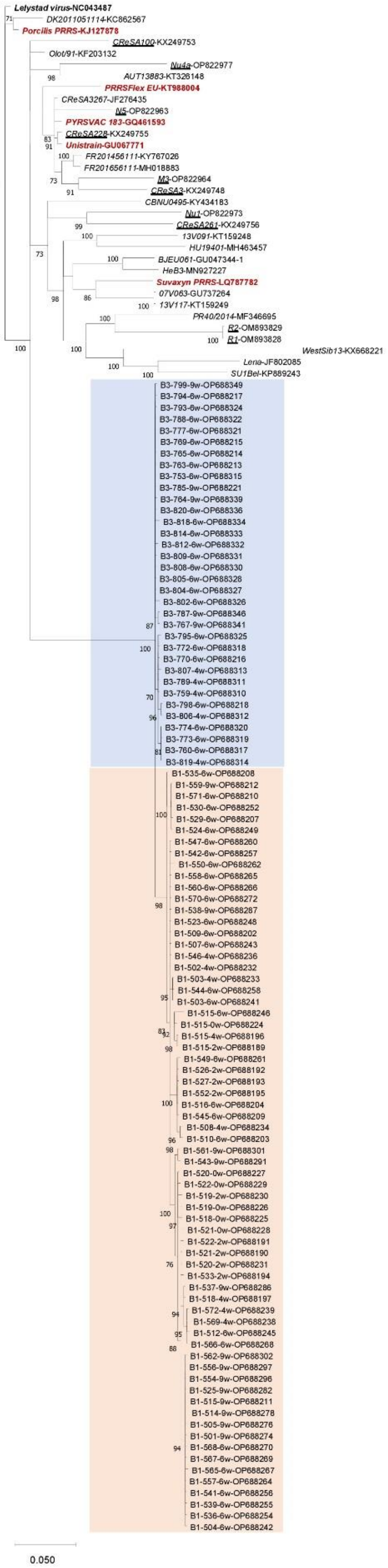

Table S3. Similarities (p-distance) between the circulating virus in this study and the reference PRRSV-1 strains. The table shows the nucleotide mean differences between the examined batches of PRRSV-1 whole genome consensus and ORF sequences and the sequences retrieved from GenBank.

	**Genome**		**ORF5**	
**PRRSV-1 strain**	Batch 1	Batch 3	Batch 1	Batch 3
***Lelystad virus*-NC043487**	0,1353	0,1374	0,1421	0,1403
Olot/91-KF203132	0,1347	0,1359	0,1484	0,1469
CReSA3-KX249748	0,1678	0,1693	0,1535	0,1521
CReSA228-KX249755	0,1347	0,1361	0,1441	0,1421
CReSA261-KX249756	0,1333	0,1341	0,1808	0,1799
CReSA100-KX249753	0,1411	0,1420	0,1720	0,1700
Nu4a-OP822977	0,1583	0,1596	0,1814	0,1799
M3-OP822964	0,1762	0,1789	0,1619	0,1601
N5-OP822963	0,1508	0,1520	0,1573	0,1568
Nu1-OP822973	0,1769	0,1776	0,1792	0,1782
R1-OM893828	0,1945	0,1953	0,1773	0,1766
R2-OM893829	0,1944	0,1952	0,1773	0,1766
CReSA3267-JF276435	0,1338	0,1350	0,1423	0,1403
AUT13883-KT326148	0,1755	0,1779	0,1611	0,1601
Lena-JF802085	0,2126	0,2138	0,2000	0,1997
SU1Bel-KP889243	0,2126	0,2131	0,1830	0,1832
07V063-GU737264	0,1643	0,1659	0,1528	0,1518
13V091-KT159248	0,1759	0,1773	0,1970	0,1980
13V117-KT159249.1	0,1644	0,1659	0,1528	0,1518
BJEU061-GU047344.1	0,1589	0,1610	0,1658	0,1667
HeB3-MN927227	0,1588	0,1605	0,1562	0,1551
DK2011051114-KC862567	0,1407	0,1427	0,1477	0,1471
FR201456111-KY767026	0,1340	0,1355	0,1475	0,1452
FR201656111-MH018883	0,1379	0,1396	0,1475	0,1452
HU19401-MH463457	0,1726	0,1737	0,2066	0,2063
PR40/2014-MF346695	0,1862	0,1859	0,1817	0,1815
WestSib13-KX668221	0,2171	0,2178	0,2111	0,2112
CBNU0495-KY434183	0,1738	0,1749	0,1646	0,1634
***Porcilis PRRS*-KJ127878**	0,1352	0,1370	0,1438	0,1436
***PRRSFlex EU*-KT988004**	0,1610	0,1625	0,1417	0,1403
***Unistrain*-GU067771**	0,1339	0,1353	0,1456	0,1436
***Suvaxyn PRRS*-LQ787782**	0,1518	0,1544	0,1665	0,1650
***PYRSVAC183*-GQ461593**	0,1341	0,1354	0,1472	0,1452

Figure S3. Bayesian phylogenetic tree based on the partial nucleotide sequences of PRRSV-1 nsp2 using MrBayes (1,000,000 iterations). nsp2 sequences obtained from born-infected animals with long viremias (in red) and the new infection cases (in black) are included. The orange and blue shaded areas correspond to sequences retrieved from Batch 1 and 3, respectively. The shaded sequences with the same color correspond to a transmission chain. Only posterior probability values >70% are shown.

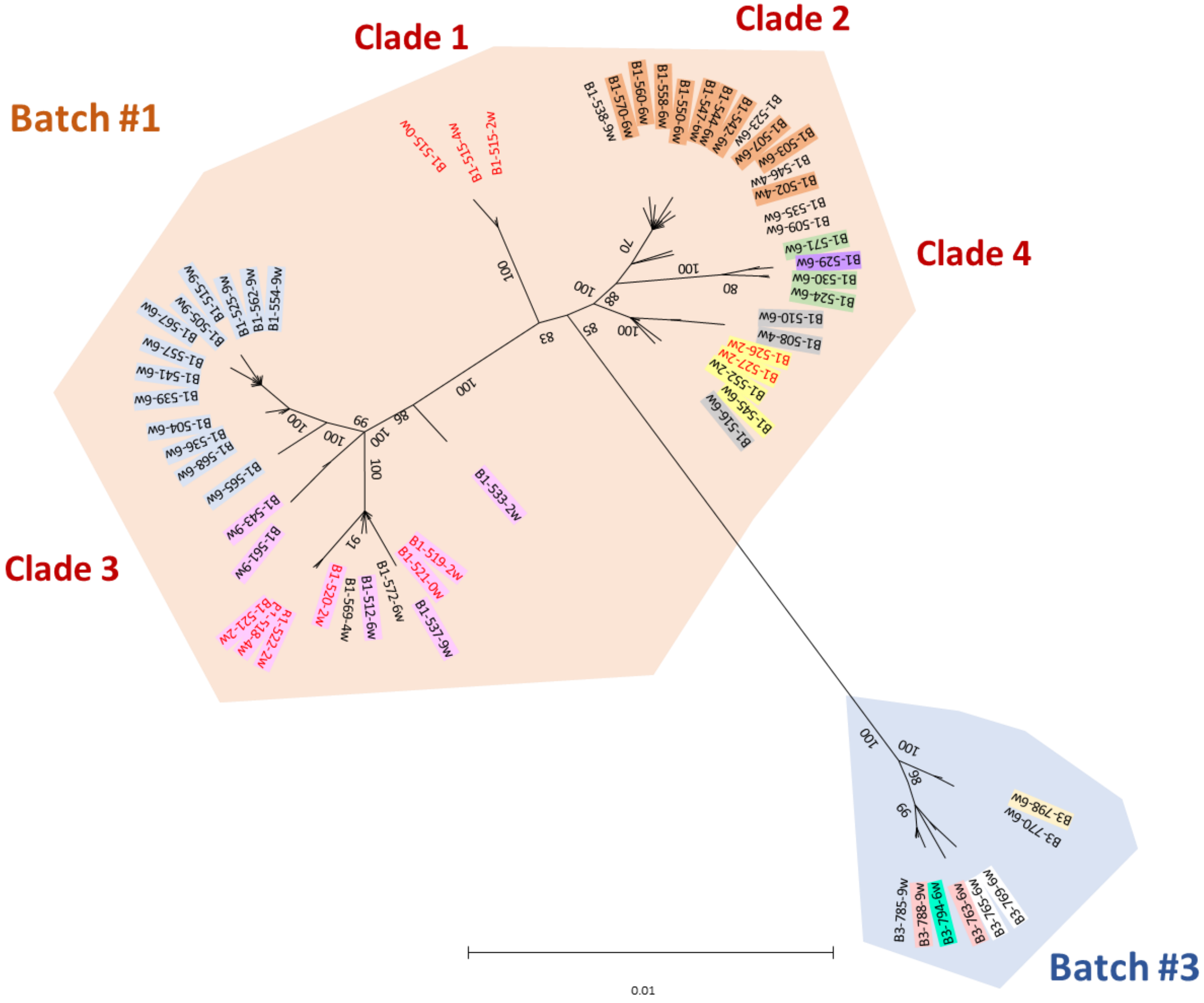

Figure S4. Bayesian phylogenetic tree based on the partial nucleotide sequences of PRRSV-1 nsp9 using MrBayes (1,000,000 iterations). nsp9 sequences obtained from born-infected animals with long viremias (in red) and the new infection cases (in black) are included. The orange and blue shaded areas correspond to sequences retrieved from Batch 1 and 3, respectively. The shaded sequences with the same color correspond to a transmission chain. Only posterior probability values >70% are shown.

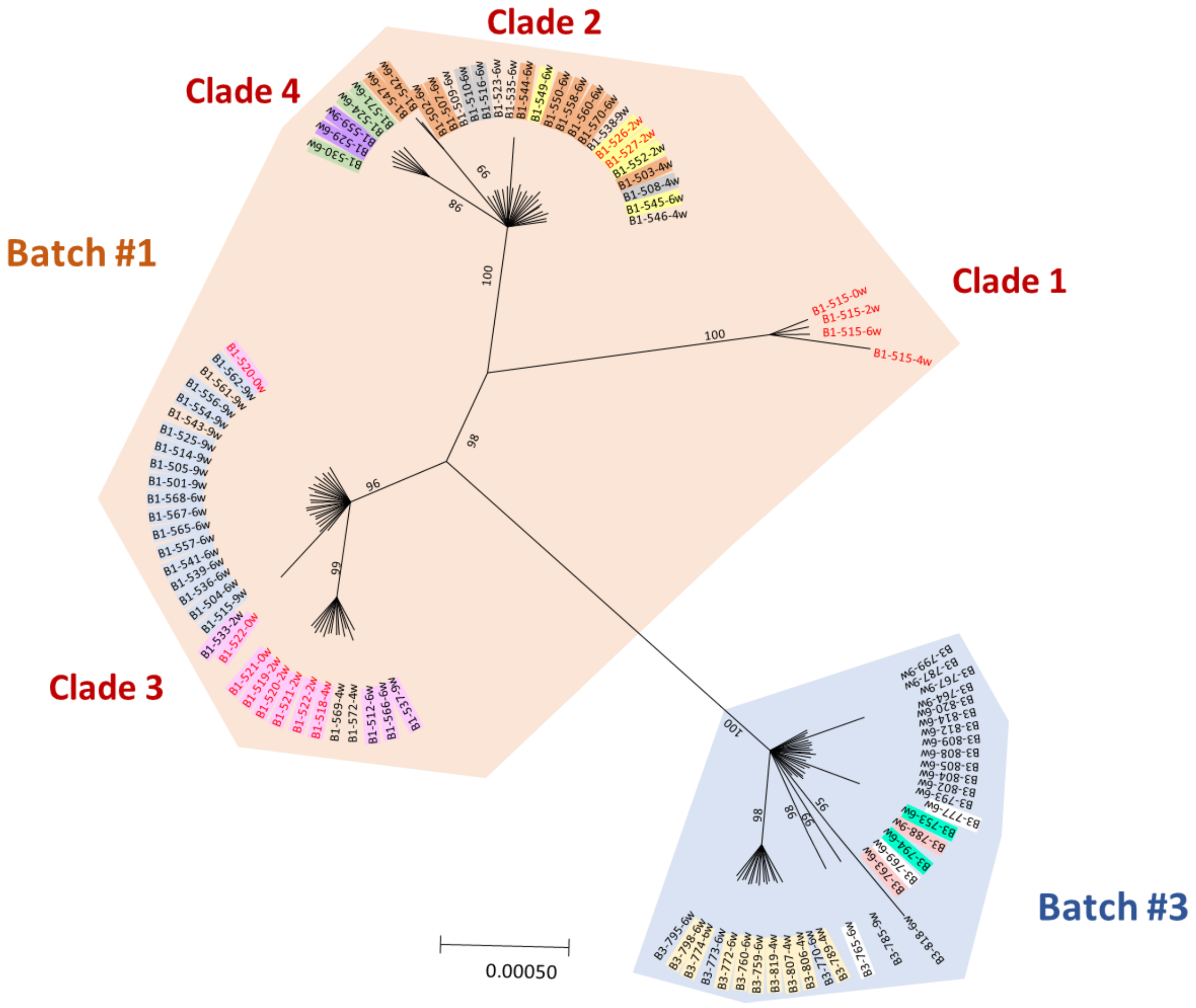

Figure S5. Bayesian phylogenetic tree based on the whole genome nucleotide sequences of PRRSV-1 using MrBayes (1,000,000 iterations). Whole genome sequences obtained from born-infected animals with long viremias (in red) and the new infection cases (in black) are included. The orange and blue shaded areas correspond to sequences retrieved from Batch 1 and 3, respectively. The shaded sequences with the same color correspond to a transmission chain. Only posterior probability values >70% are shown.

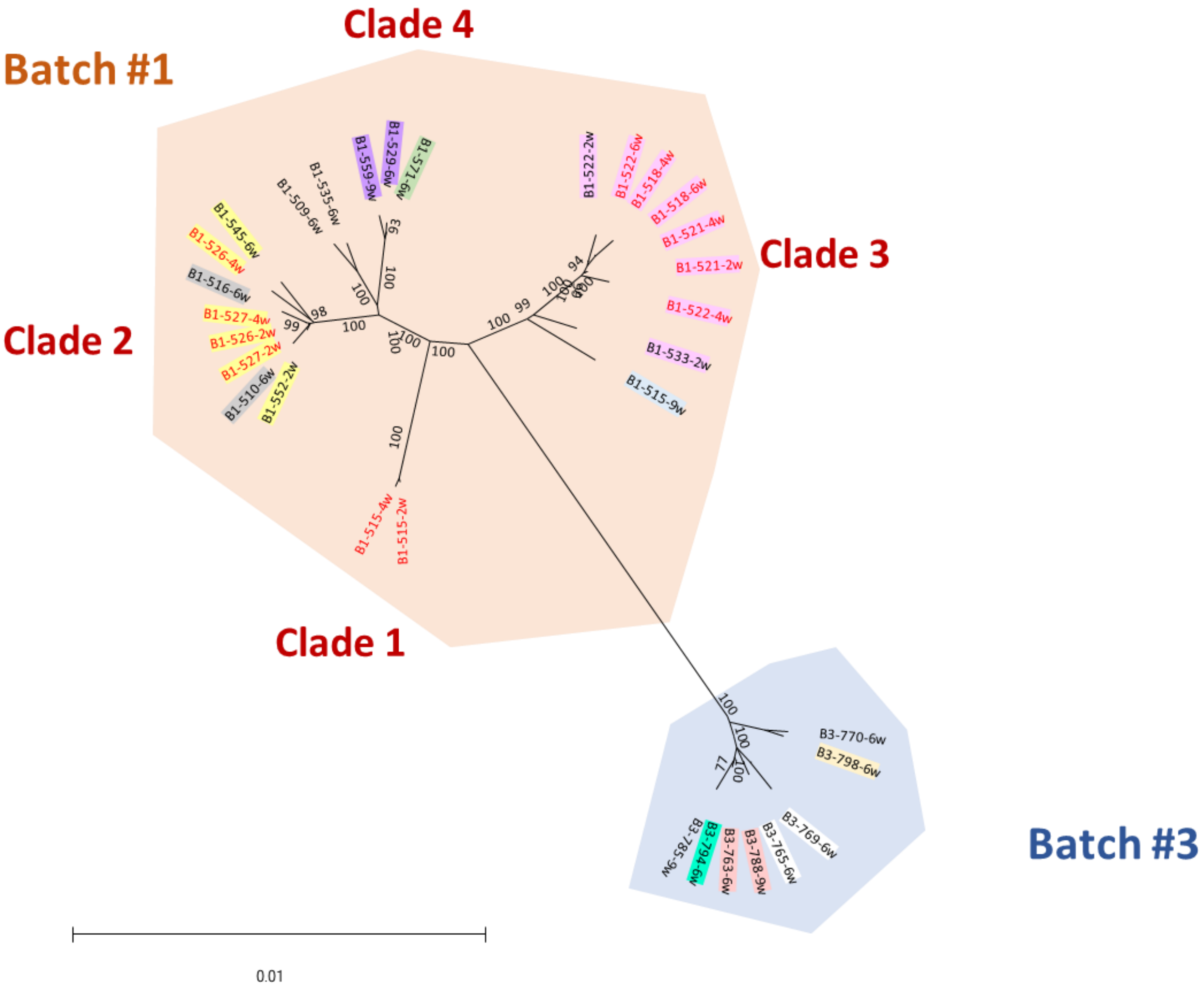

Figure S6. Maximum likelihood phylogenetic tree based on the ORF5 nucleotide sequences of PRRSV-1. Whole genome sequences obtained from born-infected animals with long viremias (in red) and the new infection cases (in black) are included. The orange and blue shaded areas correspond to sequences retrieved from Batch 1 and 3, respectively. The shaded sequences with the same color correspond to a transmission chain. Only bootstrap values >60% are shown.

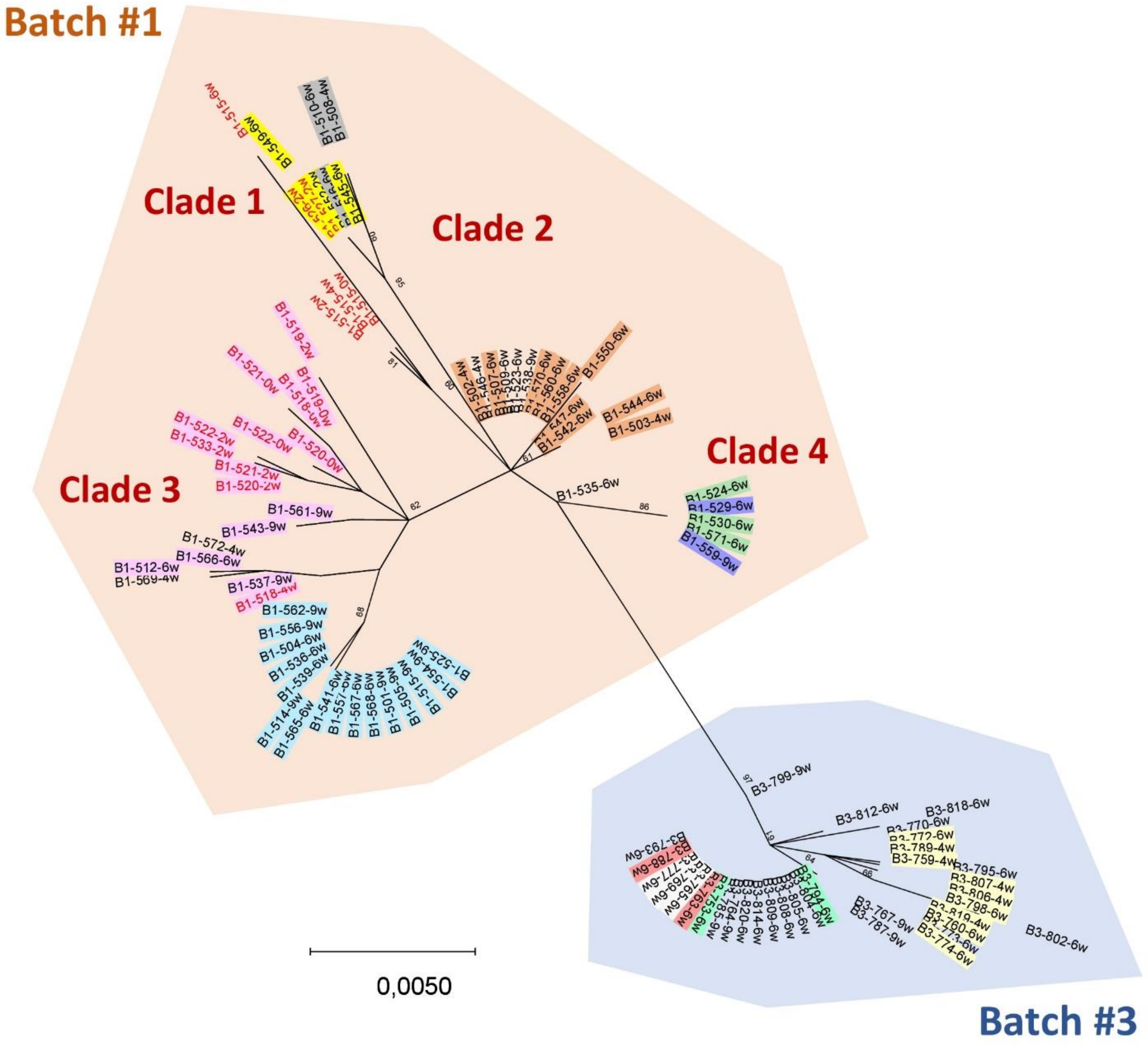

Figure S7. Maximum likelihood phylogenetic tree based on the partial nsp2 nucleotide sequences of PRRSV-1. Whole genome sequences obtained from born-infected animals with long viremias (in red) and the new infection cases (in black) are included. The orange and blue shaded areas correspond to sequences retrieved from Batch 1 and 3, respectively. The shaded sequences with the same color correspond to a transmission chain. Only bootstrap values >60% are shown.

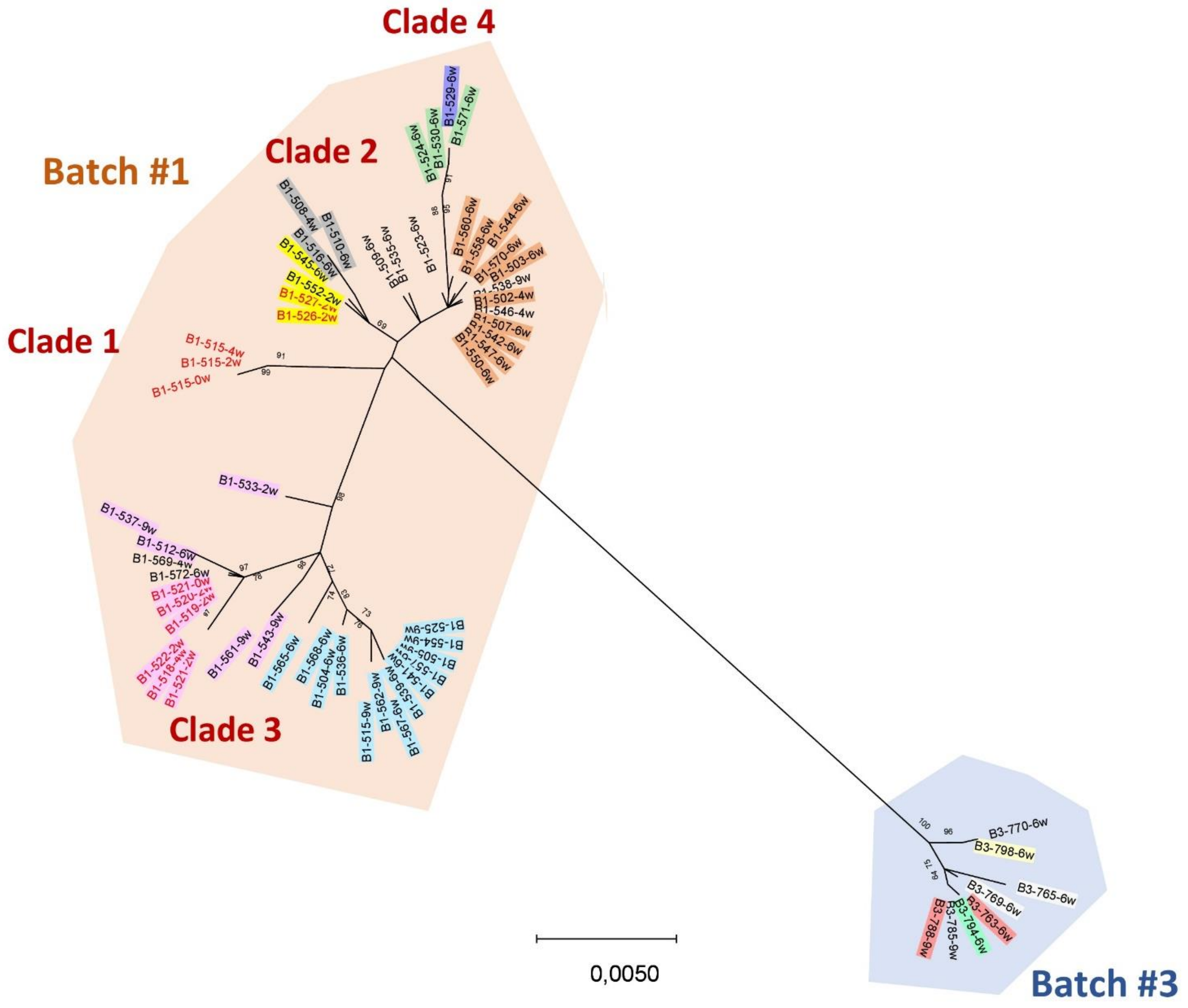

Figure S8. Maximum likelihood phylogenetic tree based on the partial nsp9 nucleotide sequences of PRRSV-1. Whole genome sequences obtained from born-infected animals with long viremias (in red) and the new infection cases (in black) are included. The orange and blue shaded areas correspond to sequences retrieved from Batch 1 and 3, respectively. The shaded sequences with the same color correspond to a transmission chain. Only bootstrap values >60% are shown.

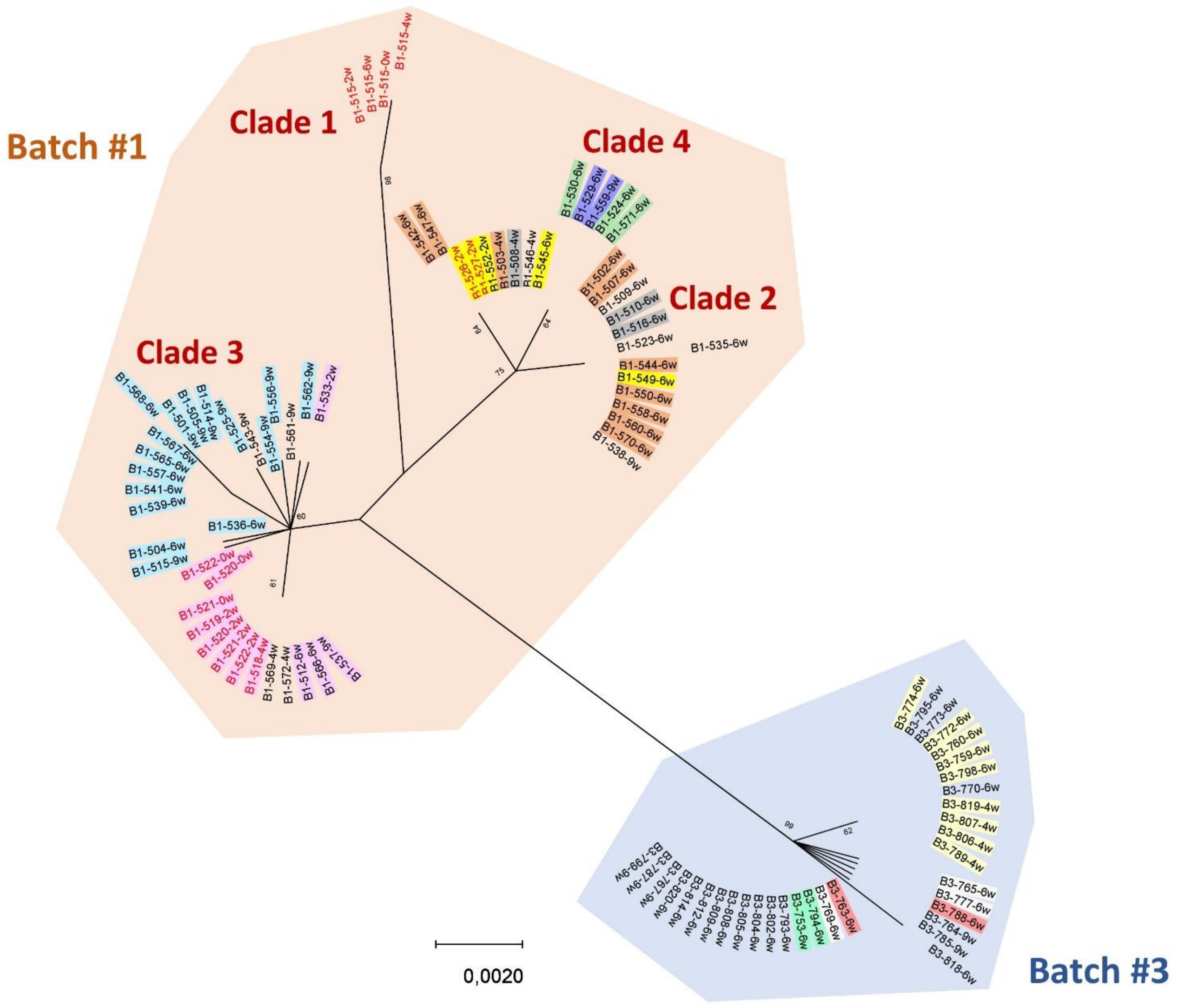

Figure S9. Maximum likelihood phylogenetic tree based on the whole genome nucleotide sequences of PRRSV-1. Whole genome sequences obtained from born-infected animals with long viremias (in red) and the new infection cases (in black) are included. The orange and blue shaded areas correspond to sequences retrieved from Batch 1 and 3, respectively. The shaded sequences with the same color correspond to a transmission chain. Only bootstrap values >60% are shown.

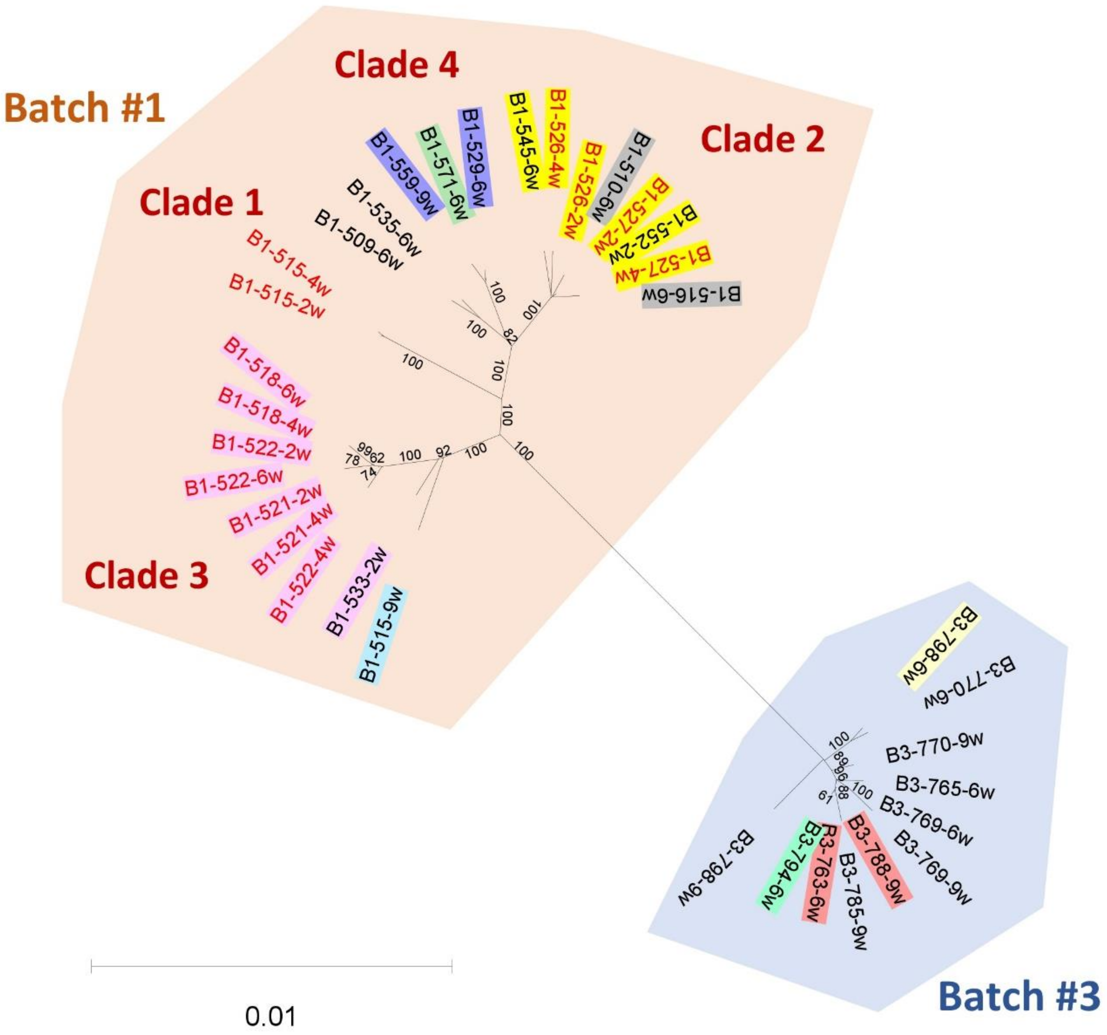

Table S4. Amino acid comparison of PRRSV-1 sequences between batches and clades. The table shows the differences between the predicted amino acid composition of the different viral proteins inferred from the consensus nucleotide sequences for each detected clade of Batch 1 (B1) and 3 (B3) (in rows). Columns indicate positions where amino acid differences were found in each examined protein. The amino acid position corresponds to the alignment with the prototype PRRSV-1 strain Lelystad (LV; NC_043487). For nsp2, all detected clades presented a 5-aa deletion between position 347-351 referred to LV. In that case, the number between brackets indicated the position considering the deletion. Grey shaded cells show the position where a different amino acid was found. Red-shaded positions indicate variations affecting known neutralizing epitopes. Changes affecting known glycosylation sites are marked with a blue circle.

	nsp1a					nsp1b								nsp2								
**Amino acid position**	43	80	127	130	151	8	16	28	72	80	81	117	178	26	174	215	269	287	291	295	303	316
**B1**-Clade1	G	T	G	P	P	K	P	G	H	H	F	G	R	Q	T	Q	P	A	P	K	E	V
**B1**-Clade2	G	T	S	P	P	E	P	G	H	H	S	D	H	Q	T	Q	P	A	P	K	E	V
**B1**-Clade3	G	V	S	P	P	E	T	G	H	H	S	G	R	K	T	Q	P	A	L	N	E	V
**B1**-Clade4	G	T	S	P	P	E	P	G	H	H	S	D	R	Q	T	R	P	A	P	K	E	V
**B3**	D	A	S	S	S	E	P	V	R	Y	S	G	R	Q	N	Q	L	T	P	K	K	M

	nsp2																	
**Amino acid position**	336	342	347-351	368 (363)	370 (365)	372 (367)	377 (372)	378 (373)	379 (374)	388 (383)	390 (385)	395 (390)	396 (391)	402 (397)	404 (399)	415 (410)	418 (413)	434 (429)
**B1**-Clade1	K	L	Del	L	S	E	S	D	W	R	A	I	T	T	G	V	Q	N
**B1**-Clade2	K	L	Del	L	S	E	N	D	W	Q	A	I	T	T	G	A	Q	S
**B1**-Clade3	K	L	Del	L	S	G	N	D	W	R	A	T	T	T	G	V	Q	S
**B1**-Clade4	K	L	Del	L	S	E	N	D	W	R	A	I	A	T	G	A	Q	S
**B3**	R	V	Del	P	P	E	N	N	R	R	T	I	T	A	S	A	R	S

	nsp2																	
**Amino acid position**	439 (434)	440 (435)	451 (446)	482 (477)	509 (504)	511 (506)	520 (515)	521 (516)	541 (536)	545 (540)	567 (562)	645 (640)	677 (672)	689 (684)	698 (693)	711 (706)	712 (707)	714 (709)
**B1**-Clade1	N	R	T	V	I	S	D	D	L	K	E	I	S	S	L	R	M	T
**B1**-Clade2	D	R	T	V	I	S	D	D	L	K	E	I	S	P	L	R	M	T
**B1**-Clade3	D	R	A	V	V	S	D	D	L	K	E	I	L	P	F	R	V	A
**B1**-Clade4	D	R	T	V	I	S	D	D	L	K	A	I	D	S	L	R	M	T
**B3**	D	G	T	I	I	F	N	G	F	T	E	T	D	P	L	C	M	T

	nsp2				nsp3			nsp4						nsp6	nsp7a	nsp9				
**Amino acid position**	719 (714)	728 (723)	830 (825)	957 (952)	59	61	77	33	72	147	163	172	188	14	145	23	106	109	160	221
**B1**-Clade1	A	V	L	S	S	S	A	T	D	D	K	L	I	S	T	E	N	I	R	V
**B1**-Clade2	A	A	L	S	P	S	A	T	E	N	R	L	I	S	T	G	D	I	R	M
**B1**-Clade3	A	A	L	S	S	S	V	I	D	N	K	L	I	N	T	E	D	T	R	M
**B1**-Clade4	T	A	L	S	P	S	A	T	E	N	R	L	V	S	T	G	D	I	R	M
**B3**	A	A	F	P	S	P	A	I	D	N	K	I	I	S	I	E	D	I	C	M

	nsp9	nsp10			nsp11				nsp12			GP2							E	GP3		
**Amino acid position**	618	12	66	428	124	139	155	182	58	59	147	8	19	78	83	197	224	236	69	18	22	30
**B1**-Clade1	Q	A	T	L	A	T	S	I	H	I	P	V	L	N	I	K	M	V	V	H	G	S
**B1**-Clade2	Q	T	T	P	A	T	S	V	H	V	P	V	L	N	V	R	I	A	I	H	S	F
**B1**-Clade3	Q	A	T	P	A	T	S	V	Y	V	P	V	L	N	V	K	I	V	V	Y	S	F
**B1**-Clade4	Q	A	T	P	A	T	S	V	H	V	P	V	P	D	V	K	I	A	V	H	S	F
**B3**	R	A	A	P	V	A	F	V	H	I	L	A	L	N	V	K	I	V	V	Y	S	F

	GP3					GP4					GP5							M					
**Amino acid position**	84	91	220	239	242	45	65	69	77	145	8	41	46	56	59	106	182	3	6	12	28	68	69
**B1**-Clade1	G	S	P	S	R	M	G	K	V	Y	E	R	D	S	S	E	V	S	D	A	M	N	R
**B1**-Clade2	G	L	P	S	H	M	S	K	V	Y	E	R	D	S	S	G	V	N	D	T	M	D	R
**B1**-Clade3	V	S	L	F	R	M	S	K	I	Y	E	R	N	S	Y	G	I	S	D	T	M	N	R
**B1**-Clade4	E	S	P	S	R	M	S	K	V	Y	E	R	N	S	S	G	V	S	D	T	M	N	R
**B3**	E	S	P	F	R	V	S	E	I	H	G	Y	N	F	S	E	V	S	G	T	I	N	Q

	M		N	
**Amino acid position**	73	130	51	127
**B1-**Clade1	I	Q	P	A
**B1-**Clade2	T	R	P	E
**B1-**Clade3	I	R	L	A
**B1-**Clade4	T	R	P	A
**B3**	T	R	P	A

Table S5. Amino acid comparison of PRRSV-1 sequences between the circulating virus and the vaccine strain used in the farm. The table shows the differences between the vaccine strain and the predicted amino acid composition of the different viral proteins inferred from the consensus nucleotide sequences of Batch 1 (B1) and 3 (B3) (in rows). Columns indicate positions where amino acid differences were found in each examined protein. The amino acid position corresponds to the alignment with the prototype PRRSV-1 strain Lelystad (LV; NC_043487). For nsp2, all detected clades presented a 5-aa deletion between position 347-351 referred to LV. In that case, the number between brackets indicated the position considering the deletion. Red-shaded positions indicate variations affecting known neutralizing epitopes.

nsp1a (n=20)

**Amino acid position**	42	43	45	47	54	57	58	80	81	100	113	117	130	138	139	140	141	148	151	162
**Vaccine**	**T**	**D**	**G**	**V**	**R**	**L**	**H**	**A**	**V**	**K**	**P**	**C**	**P**	**M**	**G**	**L**	**F**	**F**	**P**	**L**
**B1**	P	G	A	I	K	I	S	V/T	I	R	S	R	P	I	A	V	Y	S	P	S
**B3**	P	D	A	I	K	I	S	A	I	R	S	R	S	I	A	V	Y	S	S	S

nsp1b (n=57)

**Amino acid position**	1	7	8	9	15	16	17	18	21	26	28	30	33	34	37	40	42	43	44	50	58	59	62	63
**Vaccine**	**F**	**Q**	**E**	**F**	**F**	**P**	**I**	**D**	**S**	**T**	**G**	**S**	**A**	**G**	**E**	**G**	**I**	**R**	**C**	**I**	**F**	**A**	**T**	**E**
**B1**	A	R	E/K	Y	S	P/T	S	G	P	S	G	D	V	A	V	P	L	E	H	T	P	V	A	D
**B3**	A	R	E	Y	S	P	S	G	P	S	V	D	V	A	V	P	L	E	H	T	P	V	A	D

**Amino acid position**	66	67	72	77	78	80	84	86	91	94	99	102	103	104	105	106	109	110	111	114	117	118
**Vaccine**	**F**	**T**	**R**	**N**	**T**	**H**	**H**	**V**	**V**	**G**	**S**	**L**	**G**	**Q**	**S**	**A**	**R**	**C**	**H**	**H**	**D**	**A**
**B1**	L	A	H	G	V	H	Y	A	I	S	T	F	D	L	P	T	W	R	R	Y/C	G/D	S
**B3**	L	A	R	G	V	Y	Y	A	I	S	T	F	D	L	P	T	W	r	R	Y	G	S

**Amino acid position**	138	139	141	169	170	178	180	193	194	195	200
**Vaccine**	**V**	**H**	**I**	**N**	**D**	**R**	**T**	**S**	**R**	**I**	**A**
**B1**	I	N	V	D	S	R/H	M	L	P	L	V
**B3**	I	N	V	D	S	R	M	L	P	L	V

nsp2 (n=197)

**Amino acid position**	11	15	20	21	23	24	25	26	28	30	32	33	57	66	67	68	82	93	94	107	120	128	141
**Vaccine**	**A**	**E**	**P**	**T**	**K**	**V**	**A**	**L**	**V**	**T**	**G**	**I**	**I**	**T**	**Q**	**Y**	**V**	**T**	**V**	**I**	**S**	**P**	**V**
**B1**	V	A	A	A	E	A	V	Q/K	T	A	R	T	V	A	P	F	A	A	I	V	P	S	T
**B3**	V	A	A	A	E	A	V	Q	T	A	R	T	V	A	P	F	A	A	I	V	P	S	T

**Amino acid position**	143	147	148	152	155	174	179	190	200	212	215	225	238	239	242	256	262	267	269	272
**Vaccine**	**P**	**A**	**D**	**K**	**L**	**A**	**N**	**K**	**S**	**V**	**Q**	**M**	**F**	**K**	**I**	**P**	**G**	**D**	**P**	**F**
**B1**	S	Q	N	T	P	T	D	R	P	I	Q/R	V	S	E	M	S	E	A	P	S
**B3**	S	Q	N	T	P	N	D	R	P	I	Q	V	S	E	M	S	E	A	L	S

**Amino acid position**	274	276	277	279	280	281-354 (281-349)	356 (351)	357 (352)	358 (353)	359 (354)	361 (356)	362 (357)	363 (358)	364 (359)	366 (361	367 (362)	368 (363)
**Vaccine**	**P**	**S**	**P**	**R**	**P**	**Del**	**G**	**L**	**I**	**N**	**V**	**G**	**G**	**N**	**S**	**P**	**S**
**B1**	S	F	Q	K	S		N	P	V	D	T	D	R	D	P	S	L
**B3**	S	F	Q	K	S		N	P	V	D	T	D	R	D	P	S	P

**Amino acid position**	369 (364)	370 (365)	372 (367)	375 (370)	376 (371)	377 (372)	378 (373)	379 (374)	380 (375)	381 (376)	387 (382)	388 (383)	390 (385)	395 (390)	399 (394)	400 (395)
**Vaccine**	**D**	**S**	**K**	**M**	**L**	**N**	**S**	**R**	**E**	**D**	**S**	**Q**	**A**	**T**	**R**	**E**
**B1**	G	S	E/G	T	P	N/S	D	W	G	E	T	R/Q	A	I/T	G	G
**B3**	G	P	E	T	P	N	N	R	G	E	T	R	T	I	G	G

Amino acid position	402 (397)	404 (399)	405 (400)	407 (402)	409 (404)	412 (407)	414 (409)	415 (410)	418 (413)	422 (417)	424 (419)	426 (421)	427 (422)	429 (424)	432 (427)	434 (429)
**Vaccine**	**T**	**D**	**N**	**G**	**D**	**A**	**P**	**V**	**R**	**P**	**G**	**I**	**L**	**H**	**H**	**G**
**B1**	T	G	I	N	G	V	S	A/V	Q	T	K	A	P	L	R	S
**B3**	A	S	I	N	G	V	S	A	R	T	K	A	P	L	R	S

Amino acid position	435 (430)	436 (431)	437 (432)	439 (434)	440 (435)	441 (436)	442 (437)	446 (441)	448 (443)	451 (446)	452 (447)	466 (461)	482 (476)	484 (479)	488 (483)	496 (491)
**Vaccine**	**T**	**E**	**S**	**D**	**S**	**S**	**S**	**Q**	**D**	**T**	**L**	**R**	**V**	**V**	**N**	**A**
**B1**	A	G	L	D/N	R	D	L	L	V	T/A	S	K	V	A	G	V
**B3**	A	G	L	D	G	D	L	L	V	T	S	K	I	A	G	V

Amino acid position	502 (497)	507 (502)	509 (504)	511 (506)	516 (511)	519 (514)	520 (515)	521 (516)	522 (517)	535 (530)	537 (532)	539 (534)	540 (535)	541 (536)	542 (537)	544 (539)
**Vaccine**	**L**	**P**	**I**	**F**	**D**	**V**	**V**	**D**	**A**	**V**	**N**	**F**	**K**	**F**	**A**	**K**
**B1**	F	S	I/V	S	G	A	D	D	V	A	D	L	E	L	V	T
**B3**	F	S	I	F	G	A	N	G	V	A	D	L	E	F	V	T

Amino acid position	563 (558)	567 (562)	570 (565)	592 (587)	595 (590)	641 (636)	642 (637)	644 (639)	645 (640)	646 (641)	650 (645)	660 (655)	661 (656)	662 (657)	663 (658)	668 (663)
**Vaccine**	**D**	**K**	**N**	**E**	**D**	**A**	**S**	**N**	**A**	**G**	**P**	**V**	**T**	**P**	**P**	**G**
**B1**	N	E/A	S	D	N	T	G	H	I	D	L	A	I	S	S	E
**B3**	N	E	S	D	N	T	G	H	T	D	L	A	I	S	S	E

Amino acid position	670 (665)	671 (666)	673 (668)	677 (672)	678 (673)	679 (674)	680 (675)	681 (676)	684 (679)	685 (680)	686 (681)	688 (683)	689 (684)	691 (686)	694 (689)	698 (693)
**Vaccine**	**V**	**L**	**Q**	**P**	**P**	**T**	**D**	**I**	**E**	**D**	**V**	**P**	**S**	**G**	**H**	**F**
**B1**	A	P	R	S/L	L	A	G	T	K	G	I	S	S/P	K	R	L/F
**B3**	A	P	R	S	L	A	G	T	K	G	I	S	P	K	R	L

Amino acid position	699 (694)	700 (695)	701 (696)	703 (698)	704 (699)	710 (705)	711 (706)	712 (707)	714 (709)	715 (710)	716 (711)	720 (715)	723 (518)	725 (720)	727 (722)	728 (723)
**Vaccine**	**P**	**S**	**R**	**S**	**T**	**G**	**L**	**M**	**S**	**G**	**T**	**G**	**S**	**R**	**M**	**T**
**B1**	S	G	K	N	S	D	R	M/V	T/A	S	A	E	G	H	T	A/V
**B3**	S	G	K	N	S	D	C	M	T	S	A	E	G	H	T	A

Amino acid position	729 (724)	733 (728)	741 (736)	750 (745)	753 (748)	774 (769)	775 (770)	788 (783)	812 (807)	830 (825)	854 (849)	858 (853)	865 (860)	869 (864)	870 (865)	900 (895)
**Vaccine**	**W**	**V**	**M**	**S**	**P**	**I**	**L**	**L**	**N**	**L**	**V**	**K**	**Y**	**V**	**F**	**I**
**B1**	R	I	I	A	S	V	F	V	D	L	A	R	H	I	L	V
**B3**	R	I	I	A	S	V	F	V	D	F	A	R	H	I	L	V

Amino acid position	915 (910)	920 (915)	931 (026)	939 (934)	954 (949)	957 (952)	988 (983)	1004 (999)	1005 (1000)
**Vaccine**	**L**	**A**	**T**	**H**	**I**	**P**	**V**	**A**	**T**
**B1**	S	S	A	Y	V	S	I	P	V
**B3**	S	S	A	Y	V	P	I	P	V

nsp3 (n=24)

Amino acid position	18	45	54	56	59	60	61	64	77	79	83	102	129	152	159	165	174	176	179	180	195
**Vaccine**	**D**	**R**	**T**	**P**	**S**	**I**	**S**	**T**	**V**	**A**	**V**	**A**	**A**	**I**	**T**	**M**	**I**	**S**	**C**	**A**	**I**
**B1**	N	Q	I	L	P/S	A	S	A	A/V	V	I	S	T	V	V	T	V	L	G	G	V
**B3**	N	Q	I	L	S	A	P	A	A	V	I	S	T	V	V	T	V	L	G	G	V

Amino acid position	223	235	292
**Vaccine**	**I**	**I**	**V**
**B1**	V	T	I
**B3**	V	T	I

nsp4 (n=23)

Amino acid position	6	9	14	29	31	32	33	34	45	58	69	72	79	83	147	152	163	164	172	180	188	201	202
**Vaccine**	**H**	**C**	**N**	**D**	**R**	**R**	**T**	**V**	**T**	**T**	**H**	**D**	**V**	**A**	**E**	**T**	**R**	**H**	**L**	**A**	**I**	**V**	**V**
**B1**	R	S	T	G	F	F	T/1	I	A	I	N	E/D	A	E	N/D	S	K/R	Y	L	T	I/V	A	M
**B3**	R	S	T	G	F	F	I	I	A	I	N	D	A	E	D	S	K	Y	I	T	I	A	M

nsp5 (n=13)

Amino acid position	26	57	89	96	104	120	122	123	128	136	148	151	158
**Vaccine**	**I**	**A**	**V**	**I**	**S**	**L**	**M**	**T**	**T**	**T**	**C**	**N**	**S**
**B1**	V	L	I	V	P	V	V	A	I	A	H	A	A
**B3**	V	L	I	V	P	V	V	A	I	A	H	A	A

nsp6 (n=1)

Amino acid position	14
**Vaccine**	**N**
**B1**	S/N
**B3**	S

nsp7a (n=6)

Amino acid position	23	59	82	86	132	145
**Vaccine**	**N**	**I**	**I**	**I**	**L**	**T**
**B1**	D	V	T	V	P	T
**B3**	D	V	T	V	P	I

nsp7b (n=9)

Amino acid position	7	13	28	37	57	77	90	99	105
**Vaccine**	**S**	**K**	**N**	**I**	**H**	**A**	**V**	**T**	**I**
**B1**	G	R	K	V	Y	T	A	A	V
**B3**	G	R	K	V	Y	T	A	A	V

nsp8 (n=2)

Amino acid position	3	37
**Vaccine**	**K**	**L**
**B1**	R	M
**B3**	R	M

nsp9 (n=22)

Amino acid position	23	49	55	70	106	109	157	158	160	188	221	241	248	262	274	337	338	351	413	530	539	618
**Vaccine**	**E**	**V**	**T**	**I**	**D**	**I**	**H**	**K**	**R**	**H**	**M**	**K**	**Q**	**V**	**I**	**S**	**K**	**I**	**V**	**D**	**R**	**R**
**B1**	G/E	A	A	V	D/N	I/T	L	N	R	Q	M/V	T	K	I	V	T	R	V	T	E	K	Q
**B3**	E	A	A	V	D	I	L	N	C	Q	M	T	K	I	V	T	R	V	T	E	K	R

nsp10 (n=16)

Amino acid position	12	16	51	57	61	66	111	191	212	217	304	353	395	396	418	428
**Vaccine**	**A**	**Y**	**S**	**V**	**R**	**A**	**S**	**V**	**I**	**V**	**V**	**N**	**S**	**C**	**T**	**P**
**B1**	A/T	H	P	I	K	T	P	I	V	T	I	S	N	R	A	P/L
**B3**	A	H	P	I	K	A	P	I	V	T	I	S	N	R	A	P

nsp11 (n=11)

Amino acid position	23	40	74	92	119	124	139	155	161	171	182
**Vaccine**	**V**	**H**	**S**	**I**	**A**	**A**	**T**	**S**	**V**	**R**	**V**
**B1**	A	N	P	V	T	A	T	S	I	K	V/I
**B3**	A	N	P	V	T	V	A	F	I	K	V

nsp12 (n=14)

Amino acid position	58	59	83	85	97	106	115	118	120	121	127	133	147	149
**Vaccine**	**N**	**I**	**A**	**G**	**R**	**T**	**E**	**H**	**I**	**Y**	**L**	**P**	**P**	**G**
**B1**	H/Y	V/I	S	N	C	I	N	L	V	H	H	L	P	E
**B3**	H	I	S	N	C	I	N	L	V	H	H	L	L	E

GP2 (n=35)

Amino acid position	2	5	8	13	14	19	27	29	30	39	41	42	47	48	59	74	78	83	88	95	112	114	129
**Vaccine**	**Q**	**Y**	**V**	**C**	**S**	**L**	**I**	**L**	**F**	**P**	**Q**	**D**	**S**	**F**	**L**	**S**	**N**	**F**	**L**	**I**	**R**	**R**	**S**
**B1**	R	H	V	Y	L	L/P	T	S	S	Q	P	A	F	Y	R	G	N/D	L	F	V	Q	Q	A
**B3**	R	H	A	Y	L	L	T	S	S	Q	P	A	F	Y	R	G	N	L	F	V	Q	Q	A

Amino acid position	138	141	184	193	194	197	199	206	224	236	246	248
**Vaccine**	**G**	**I**	**H**	**G**	**T**	**K**	**T**	**I**	**I**	**V**	**H**	**S**
**B1**	S	V	R	S	S	K/R	A	V	I/M	A/V	R	L
**B3**	S	V	R	S	S	K	A	V	I	V	R	L

E (n=4)

Amino acid position	3	47	54	69
**Vaccine**	**L**	**L**	**L**	**V**
**B1**	S	F	I	V/I
**B3**	S	F	I	V

GP3 (n=45)

Amino acid position	3	4	7	9	11	12	14	15	16	18	22	30	31	48	58	64	79	84	91	93	99	135	154
**Vaccine**	**H**	**Q**	**R**	**H**	**F**	**L**	**G**	**F**	**I**	**Y**	**S**	**S**	**T**	**M**	**S**	**R**	**Y**	**E**	**L**	**P**	**D**	**F**	**V**
**B1**	C	K	C	Y	L	F	S	I	V	H/Y	S/G	F/S	A	K	L	E	H	G/V/E	S/L	F	E	Y	I
**B3**	C	K	C	Y	L	F	S	I	V	Y	S	F	A	K	L	E	H	E	S	F	E	Y	I

Amino acid position	157	158	166	186	204	214	216	220	222	230	232	235	236	239	242	245	246	251	254	255
**Vaccine**	**G**	**H**	**A**	**L**	**V**	**I**	**R**	**P**	**L**	**T**	**I**	**D**	**L**	**S**	**R**	**K**	**F**	**R**	**V**	**V**
**B1**	E	R	V	F	A	T	K	P/L	V	K	N	N	I	S/F	R/H	P	V	H	A	A
**B3**	E	R	V	F	A	T	K	P	V	K	N	N	I	F	R	P	V	H	A	A

Amino acid position	256	257
**Vaccine**	**K**	**P**
**B1**	R	L
**B3**	R	L

GP4 (n=25)

Amino acid position	5	15	35	41	45	49	51	54	55	57	60	61	63	64	65	67	69	70	77	109	115	123	145	163	166
**Vaccine**	**T**	**I**	**E**	**A**	**M**	**D**	**N**	**R**	**P**	**G**	**A**	**A**	**E**	**E**	**I**	**F**	**K**	**S**	**V**	**H**	**G**	**W**	**H**	**T**	**A**
**B1**	I	L	K	G	M	K	E	Q	Y	R	T	T	K	A	S/G	L	K	P	V/I	Y	E	G	Y	S	V
**B3**	I	L	K	G	V	K	E	Q	Y	R	T	T	K	A	S	L	E	P	I	Y	E	G	H	S	V

GP5 (n=38)

Amino acid position	2	4	6	8	15	17	20	22	41	46	56	59	60	63	71	75	79	89	90	100	104	106	111
**Vaccine**	**R**	**S**	**K**	**G**	**S**	**F**	**F**	**L**	**Y**	**N**	**D**	**S**	**S**	**G**	**F**	**A**	**L**	**F**	**F**	**T**	**V**	**G**	**C**
**B1**	K	F	R	E	F	C	L/P/F	F	R	D/N	S	S/Y	G	D/N	L	I	I	L	L	T/I	I	G/E	S
**B3**	K	F	R	E	F	C	L	F	Y	N	F	S	G	D	L/F	I	I	L	L	T/I	I	E	S

Amino acid position	116	119	122	123	125	126	150	154	162	171	172	173	182	195	201
**Vaccine**	**A**	**F**	**F**	**V**	**F**	**V**	**N**	**V**	**V**	**V**	**D**	**G**	**V**	**S**	**A**
**B1**	V	L	L	I	L/F	A	D	I	L	I/V	G	S	I/V	P	A/T
**B3**	V	L	L	I	L	A	D	I	L	I	G	S/N	V	P	A

M (n=14)

Amino acid position	3	6	9	11	12	28	62	65	68	69	72	73	125	130
**Vaccine**	**G**	**D**	**N**	**P**	**I**	**I**	**V**	**Q**	**N**	**R**	**F**	**T**	**S**	**R**
**B1**	S/N	D	G	S/P	T/A	M	A	H	N/D	R	L	T/I	P	R/Q
**B3**	S	G	G	S	T	I	A	H	N	Q	L	T	P	R

N (n=13)

Amino acid position	13	22	32	33	35	43	48	49	51	69	100	127	128
**Vaccine**	**S**	**P**	**A**	**M**	**K**	**G**	**K**	**K**	**P**	**T**	**S**	**A**	**S**
**B1**	N	S	S	V	R	R	R	N	P/L	N	G/S	A/E	N
**B3**	N	S	S	V	R	R	R	N	P	N	G	A	N

Figure S10. S/P ratio values of the piglets by parity of the sow as determined by ELISA. Each dot represents an examined individual. S/P ratio values ≥0.4 are considered positive. The graphs show the S/P ratio values of the piglets at 2 weeks of age by parity of the sow in Batch 1 (**A**), Batch 2 (**B**), and Batch 3 (**C**).

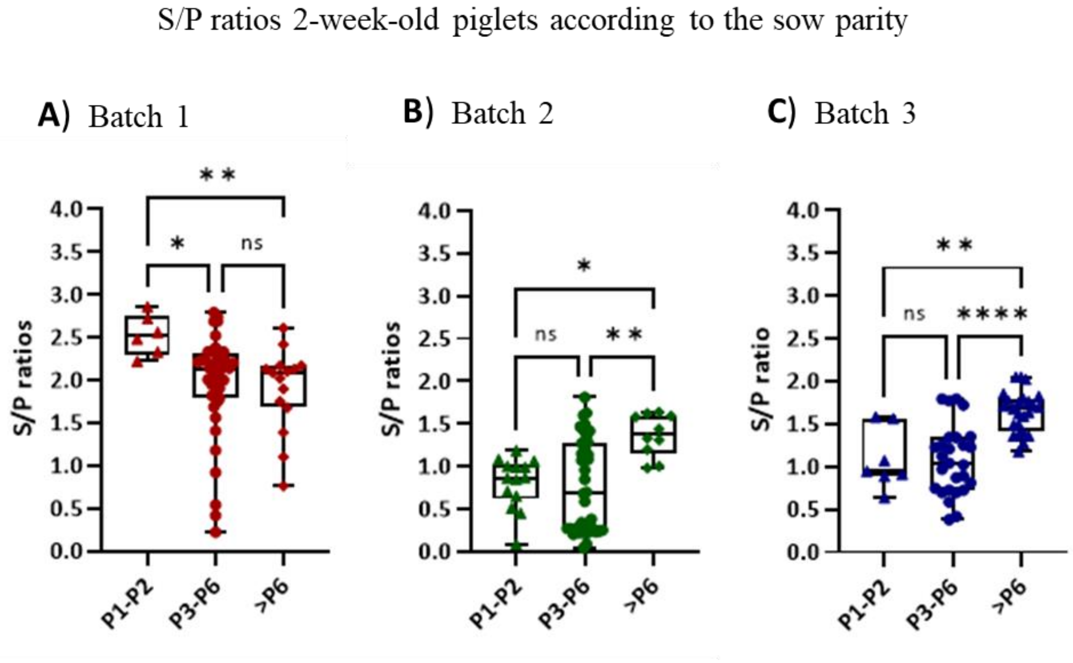

Supplement: Supplementary file 1 [file Data_Sheet_1.PDF]
